# Mechanism of a Halogen Exchange Reaction in Water: Catalysis by Aqueous Media

**DOI:** 10.1021/acscentsci.4c02228

**Published:** 2025-03-04

**Authors:** Imon Mandal, Itai Zakai, Natalia V. Karimova, Mark A. Johnson, R. Benny Gerber

**Affiliations:** † The Fritz Haber Center for Molecular Dynamics, Institute of Chemistry, The Hebrew University of Jerusalem, Jerusalem 91904, Israel; ‡ Department of Chemistry, University of California, Irvine, California 92697, United States; § Sterling Chemistry Laboratory, Department of Chemistry, Yale University, New Haven, Connecticut 06520, United States

## Abstract

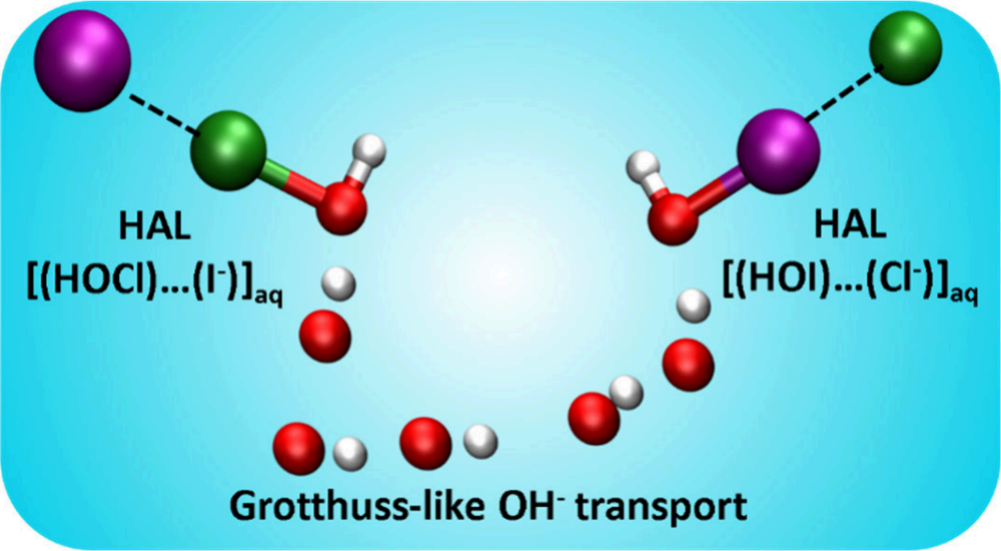

Reactions of Cl^–^, Br^–^, and I^–^ ions in seawater with incoming molecules from the gas phase are of major atmospheric importance, but their mechanisms are mostly unknown. In this study, using ab initio molecular dynamics (AIMD) simulations, the microscopic mechanism of the halogen exchange reaction in water, HOCl + I^–^ → HOI + Cl^–^, is unraveled. The main findings are as follows: (1) The reaction proceeds through a halogen-bonded isomer of the complex of HOCl with I^–^, which is present in water and has a significant lifetime. The hydrogen-bonded isomer of the complex seems to play no role in the reaction. (2) Several water molecules act to catalyze the reaction through a Grotthuss-like mechanism that is totally different from that of halogen exchange in the gas phase. These results may have important implications for the chemistry of seawater, in particular for other reactions involving halogenated species.

## Abstract 2

Halogen exchange between iodide ions and hypochlorous acid molecules in seawater is catalyzed by the medium through the formation and participation of hydroxyl ions.

Processes at the surface of seawater have major effects on the chemistry of the atmosphere. One such process of interest here involves reactions between halide anions present in water, Cl^–^, Br^–^_,_ and I^–^, with incoming molecules from the gas phase. In this Article, we focus on reactions, hitherto not understood, between hypohalous acids (HOX) and halide ions in the water. The background for the importance of heterogeneous reactions in atmospheric chemistry is as follows. The seminal discovery of Rowland and Molina describing ozone depletion by free halogens^1^ and the role of halogens as oxidizers of hydrocarbons have inspired a substantial amount of work directed at unraveling the origins of halogens in the atmosphere.^2−11^ These studies established that the halogen-containing molecules HOX, HX, X_2_, and XNO_2_ (where X= Cl, Br, I) are the main reservoirs of halogens in the stratosphere and troposphere. HOXs lead to the production of dihalogens in the presence of halides (Y^–^, where Y = Cl, Br, I) at the water–air interface^12−14^ through the following mechanism: the HOX molecule is taken up by water surfaces and reacts with Y^–^ to release a gaseous XY molecule and a solvated OH^–^ ion.^4,5,7,15,16^ This reaction is greatly enhanced under low-pH conditions.^16−20^ The XY molecule, itself a pollutant, is subsequently photolyzed to produce the ozone-depleting X and Y.^5,15,16^ The key atmospheric importance of XY formation from HOX + Y^–^ naturally brings attention to processes that compete with this reaction. So far, the possibility of the competing halogen exchange reaction,^21^ which leads from HOX and Y^–^ to HOY and X^–^ in water, has remained unexplored. This process may potentially affect the mixing ratios of different HOX species in aerosols and on water surfaces.

Among the halogens, the main contributor to halogen-related surface ozone destruction is iodine.^9^ In certain regions of the globe, tropospheric iodine levels have tripled since the mid-20th century,^22,23^ highlighting the need for a comprehensive understanding of iodine sources in the atmosphere. The main origin of the atmospheric iodine comes from oceanic emissions, with around 80% arising from abiotic sea-air emissions of inorganic iodine in the form of molecular iodine (I_2_) and hypoiodous acid (HOI).^9,11^ Notably, HOI is emitted at a rate ten times higher than I_2_ under ambient conditions.^9^ Thus, thorough knowledge about the sources, distribution, and fate of HOI is essential to establish the role of iodine species in the atmospheric chemistry. Because halogen exchange offers a new potential pathway for HOI formation from other HOXs (X = Cl, Br), it is useful to explore the mechanism of this reaction under conditions relevant to the air–water interface region of seawater. We use HAL[(HOX)···(Y^–^)]_aq_ and HYD[(HOX)···(Y^–^)]_aq_ as the notation for the halogen- and hydrogen-bonded complexes in water. When subscript aq is not used, the notation refers to the gas-phase complexes. [(HOX)···(Y^–^)] refers to both gas-phase halogen- and hydrogen-bonded complexes. Insights into the halogen exchange reaction were recently provided by Johnson and co-workers, who explored the structure and stability of dry gas-phase complexes of [(HOCl)···(I^–^)].^21,24^ They captured and structurally characterized a HAL[(HOI)···(Cl^–^)] complex formed upon the uptake of HOCl vapor by I^–^·(H_2_O)_*n*_ clusters in a radiofrequency ion guide via vibrational spectroscopy. The gas-phase calculations indicated that the halogen exchange reaction HOCl + I^–^ → HOI + Cl^–^ occurs in multiple steps with high barriers. It was also suggested that more efficient conversion of HOCl and I^–^ to HOI and Cl^–^ could occur through pathways involving the water molecules that are present in the preparation of the complexes. However, the mechanism of the reaction in aqueous media remains unknown.

In this Article, we unravel the microscopic mechanism of the halogen exchange reaction HOCl + I^–^ → HOI + Cl^–^ in water by calculating ab initio molecular dynamics trajectories of the pre-reactive complexes of [(HOX)···(Y^–^)]_aq_ (X, Y = Cl, I, Cl) at a water slab. As the HOX encounters Y^–^ anions, entrance channel complexes may form via either a halogen bond between X and Y^–^, i.e., the HAL[(HOX)···(Y^–^)]_aq_ complex,^21^ or a hydrogen bond between H and Y^–^, i.e., the HYD[(HOX)···(Y^–^)]_aq_ complex,^24^ as reported earlier in the case of dihalogen formation.^19^ The structures and lifetimes of the two types of complexes are relevant to the reaction dynamics, and we analyze these results with a detailed description of the halogen exchange mechanism. A specific goal of this work is to establish whether HAL[(HOCl)···(I^–^)]_aq_ complexes play an important role in the halogen exchange reaction and to elucidate the contribution that water plays in facilitating this transformation.

## Results and Discussion

### Structures and Lifetimes of [(HOX)···(Y^–^)] Complexes in Water (X, Y = Cl, I or I, Cl)

We start our discussion of the halogen exchange reaction in water from the formation of the [(HOX)···(Y^–^)]_aq_ pre-reactive complexes, where X, Y= Cl, I or I, Cl. The stabilities and lifetimes of these pre-reactive complexes are key features of the reaction mechanism, as they allow the two reagents to interact for prolonged periods before they react. In total, we have studied the structures and lifetimes of four different complexes, two of which are HAL[(HOCl)···(I^–^)]_aq_ and HAL[(HOI)···(Cl^–^)]_aq_ and the other two of which form the respective hydrogen-bonded complexes.

**Figure 1:**
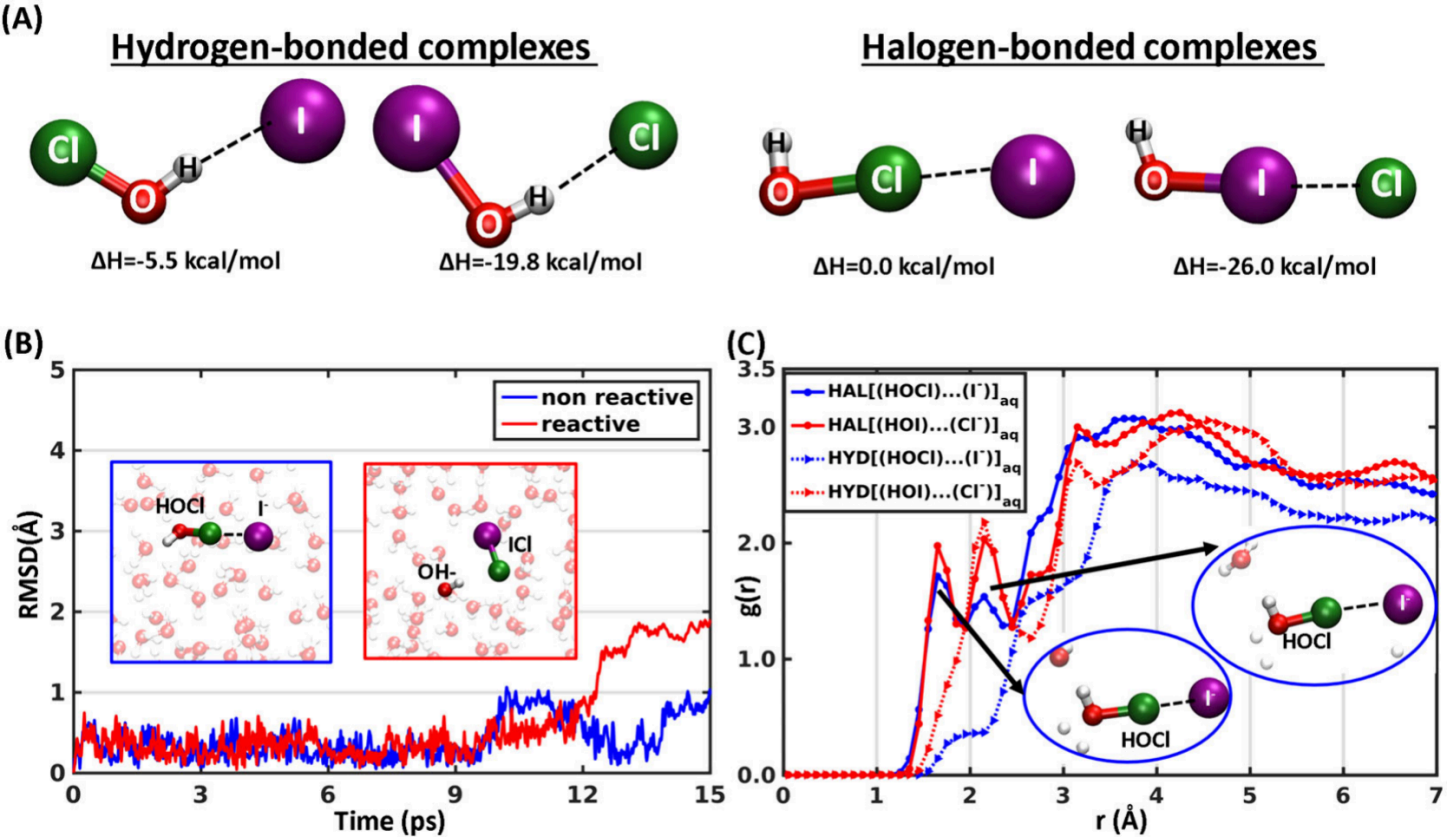
Stability of pre-reactive complexes. (A) Gas-phase optimized structure (MP2/aug-cc-pVTZ) of the hydrogen- and halogen-bonded pre-reactive complexes of [(HOX)···(Y^–^)] (X, Y = Cl, I or I, Cl)(adopted from ref (21)) and their enthalphy in kcal/mol(1 kcal/mol = 4.184 kJ/mol). (B) RMSD of the HAL[(HOCl)···(I^–^)]_aq_ complexes (four atoms H, O, Cl, and I) along the time trajectory showing the complexes are stable at least 15 ps except the reactive ones. The non reactive legend indicates one representative trajectory among ten trajectories that remain unreactive during simulations. The reactive trajectory represents one of the five that reacted. Snapshots of the fate of the HAL[(HOCl)···(I^–^)]_aq_ complexes at time 15 ps for representative unreactive and reactive simulations are shown in the inset with colored boundaries. (C) Radial distribution plot of [(HOX)···(Y^–^)] complexes with the all other atoms present in the slab. Representative snapshots of the first and second solvation shells with water hydrogens and oxygen for HAL[(HOCl)···(I^–^)]_aq_ are provided in the inset.

To study the complexes in water, we have employed an ab initio model of liquid water with periodic boundary conditions, as described in detail in the Methods section. All simulations retain the halogen- and hydrogen-bonded [(HOX)···(Y^–^)]_aq_ pre-reactive complexes in water (Figure 1 and Figure S1). We adopt geometrical criteria to determine the formation and breaking of halogen and hydrogen bonds, as mentioned in our previous work: an H–Y^–^ distance smaller than 3.2 Å (1 Å = 1× 10^–10^ m) and an ∠O–H···Y^–^ angle larger than 140° were chosen as hydrogen bond formation criteria, and a halogen bond is formed when the X–Y^–^ distance is smaller than 3.5 Å and the ∠Y^–^···X–O angle is larger than 130°.^19^ To obtain an overall structural measure to track the evolution of the complex structure along an MD trajectory with respect to a reference structure (here a gas-phase structure), we have plotted the root-mean-square deviation (RMSD) of the 4 atoms of H, O, X, and Y^–^ of [(HOX)···(Y^–^)]_aq_ complexes. Figure 1B and Figure S1 depict the variation of the behavior of the complexes in water from the corresponding gas-phase species. Stabilization in the RMSD of the four atoms of the complexes signifies a stabilized complex structure. These figures indicate the following results: (a) all trajectories (except the events of forming solvent separated molecule ion pair) for [(HOX)···(Y^–^)]_aq_ complexes do not dissociate in water for at least 15 ps (our simulation time). This stability does not pertain to the trajectories where the reaction occurs; there, the complexes start reacting after ∼5 ps. (b) [(HOX)···.(Y^–^)]_aq_ complexes in both hydrogen- and halogen-bonded forms are similar to the initial gas-phase structures. For both HYD[(HOCl)···(I^–^)]_aq_ and HYD[(HOI)···(Cl^–^)]_aq_ complexes, the fluctuations in RMSD are up to 3.5 Å due to either the competition between Y^–^ and surrounding waters (Figures S1C and D) for a HOX hydrogen bonding partner or due to the formation of a solvent-separated HOX and Y^–^ molecule–ion pair. Formation of the solvent-separated pairs is suggested by larger Y^–^–H distances (Figure S2). Interestingly, the stable hydrogen-bonded complexes remain unreactive during simulations (Figure S3). The RMSD fluctuations of HAL[(HOCl)···(I^–^)]_aq_ and HAL[(HOI)···(Cl^–^)]_aq_ complexes, on the other hand, are ∼1 Å, except for the trajectories in which the HAL[(HOCl)···(I^–^)]_aq_ moiety initiates the halogen exchange reaction (Figure 1 and Figure S1A and B). The neighboring water molecules provide structural stabilization of the halogen-bonded complexes. The radial distribution function (RDF) plots, denoted as *g*(*r*) in Figure 1C, of different [(HOX)···(Y^–^)]_aq_ complexes, which are proportional to the number of molecules within a spherical shell of radius *r* centered around the complex and averaged over all time-frames, reinforce this stabilization. Figure 1C shows that HAL complexes have two structured solvation layers below 3 Å indicated by two peaks, whereas the HYD complexes, specially HYD[(HOCl)···(I^–^)]_aq_, lack any structured solvation shell. The gas-phase calculations reveal the following order of stability: HAL[(HOI)···(Cl^–^)] (−25.96 kcal/mol) > HYD[(HOI)···(Cl^–^)] (−19.81 kcal/mol) > HYD[(HOCl)···(I^–^)] (−5.49 kcal/mol) > HAL[(HOCl)···(I^–^)] (0.0 kcal/mol).^21^ In the presence of water, both HAL[(HOCl)···(I^–^)]_aq_ and HAL[(HOI)···(Cl^–^)]_aq_ complexes exhibit lifetimes of at least ∼5 ps (obtained from the stabilization in the RMSD). Subsequently, some HAL[(HOCl)···(I^–^)]_aq_ complexes started reacting but HAL[(HOI)···(Cl^–^)]_aq_ remains stable throughout the simulation time (Figure 1 and Figure S1).

Although the role of halogen bonding in chemistry is much less explored than hydrogen bonding, these bonds can play a major role in reactions.^19^ It was only in the 1990s that the physics underlying halogen bonding was understood through computer simulations.^25^ These simulations predicted that an electron-withdrawing group in a molecule that contains a halogen shifts the electron density on the halogen atom and produces a low electron density region on it. Thus, the halogen atom may serve as an electrophile and form a halogen bond in the presence of a nucleophile. Because the strength of the bond depends on the shifting of the electron density, halogens with larger polarizabilities donate stronger halogen bonds.^25^ Hence, HOI is expected to form a stronger halogen bond with halides than HOCl as obtained both in the gas phase and with water slab simulations.

In summary, here we shed light on the crucial role of HAL[(HOCl)···(I^–^)]_aq_ complexes in an emerging halogen exchange reaction. Our simulations provide compelling evidence that HAL[(HOCl)···(I^–^)]_aq_ complexes persist for a sufficient duration in water to serve as crucial precursors to the halogen exchange reaction, as shown in the next section. HYD[(HOX)···(Y^–^)]_aq_ (X,Y= Cl,I or I, Cl) complexes do not show any halogen exchange reaction.

### The Halogen Exchange Reaction HOCl + I^-^ → HOI + Cl^-^ in Water

The halogen exchange reaction proceeds from the least stable HAL[(HOCl)···(I^–^)]_aq_ to more stable HAL[(HOI)···(Cl^–^)]_aq_. Hence, the reaction is exothermic. The reaction initiated in five out of fifteen simulations, and we extended those to 50 ps to enable analysis of all the processes involved in the overall mechanism. Investigation of the reacting trajectories shows the following qualitative mechanism involving three sequential steps:

(1) ICl formation: HAL[(HOCl)···(I^–^)]_aq_ → ICl + OH^–^,
(2) Migration of OH^–^ through proton relay in water molecules by a Grotthuss-like mechanism: OH^–^ + H_2_O → H_2_O + OH^–^,
(3) Reaction of ICl and OH^–^ (attacking from the I side) to form the HAL[(HOI)···(Cl^–^)]_aq_ complex.

**Figure 2:**
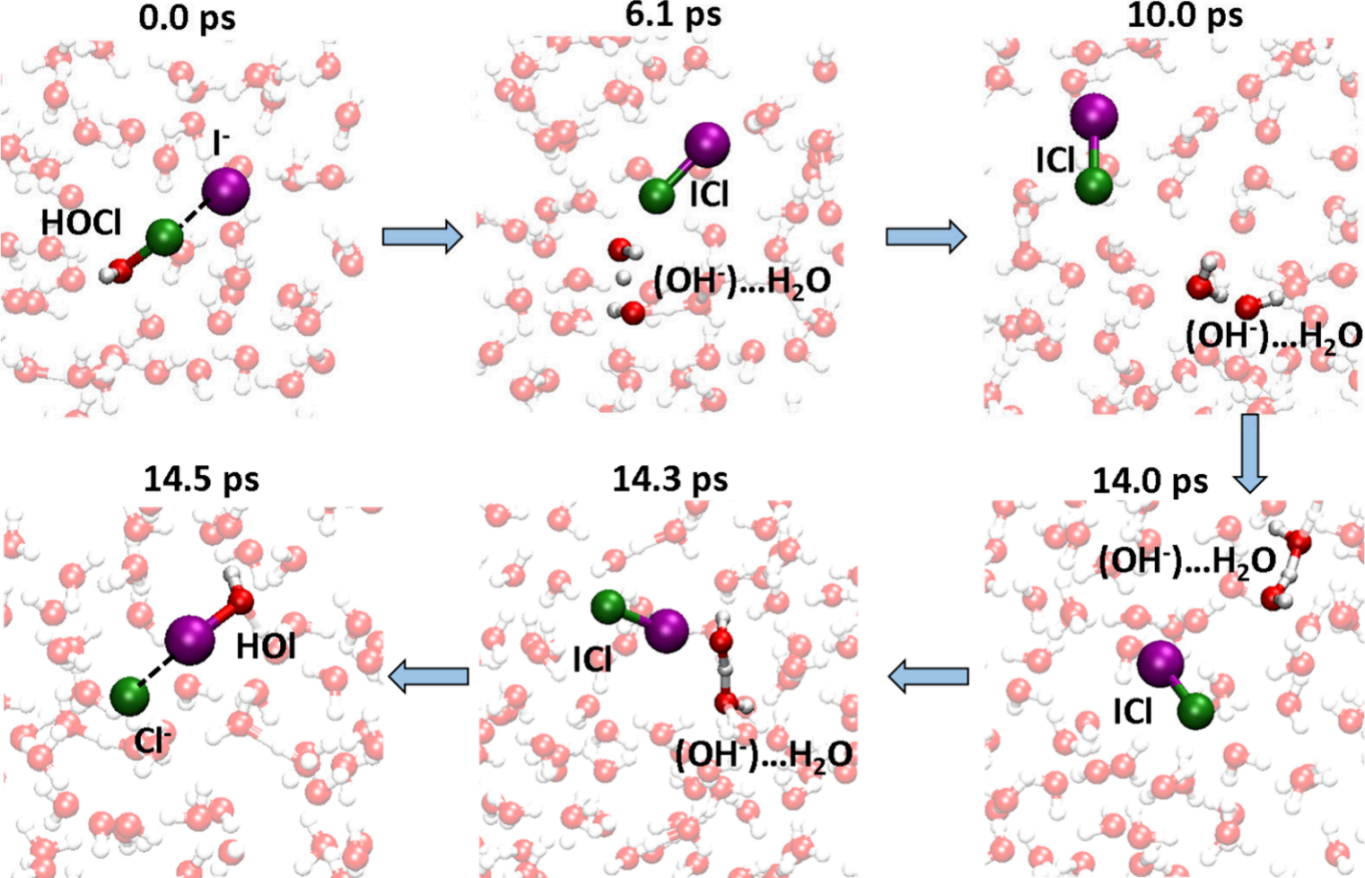
Halogen exchange reaction mechanism. Snapshots for the multiple steps involved in the halogen exchange reaction HAL[(HOCl)···(I^–^)]_aq_ to HAL[(HOI)···(Cl^–^)]_aq_.

**Figure 3:**
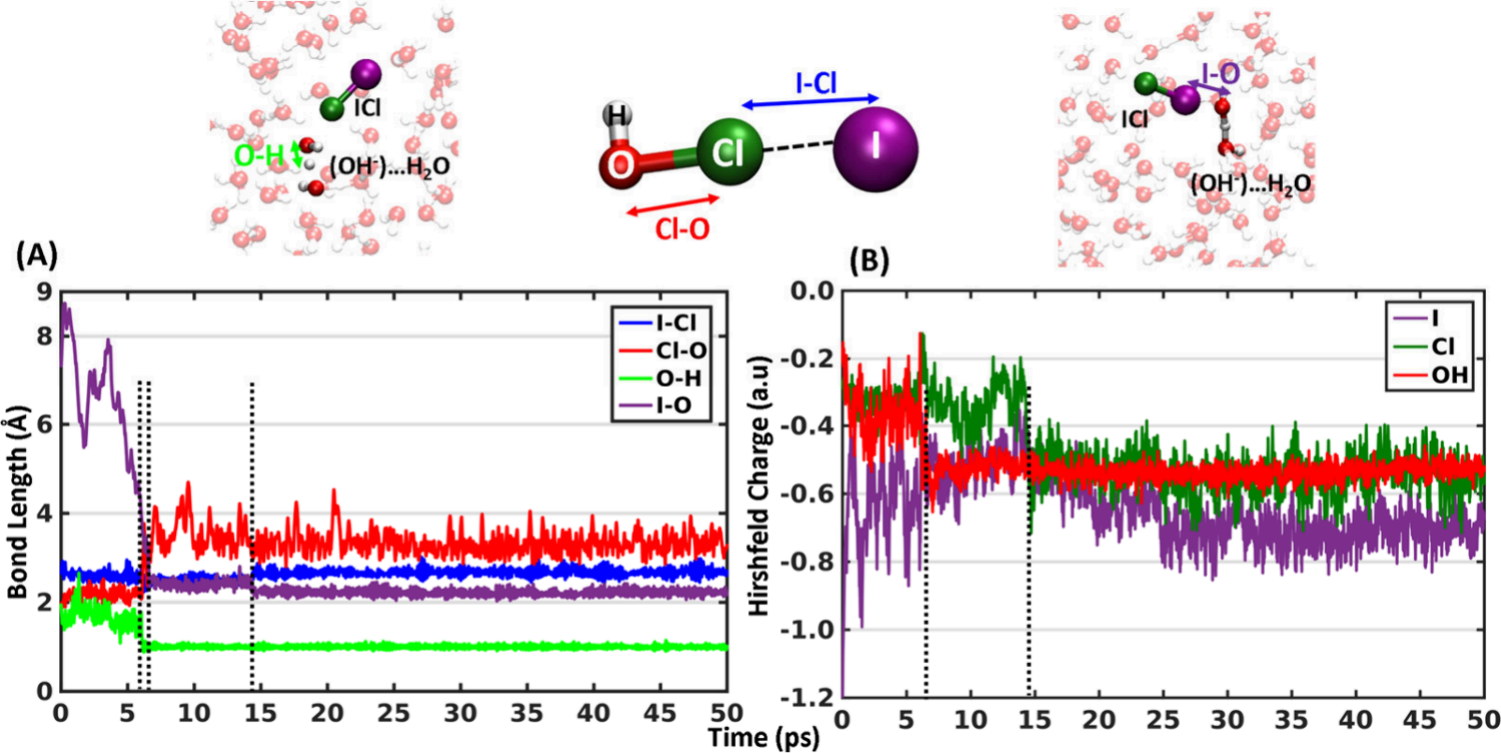
Bond length and partial charge evolution during the complete halogen exchange reaction. Time evolution of the (A) bond lengths (I–Cl, Cl–O, O–H, and I–O) and (B) Hirshfeld partial charges (on I, Cl, summation of O and H from HOCl) along the trajectory where complete halogen exchange occurs. The vertical black dotted lines in parts A and B are eye guides for the events (mentioned in the text) involved in the halogen exchange reaction. Structures of the HAL[(HOCl)···(I^–^)]_aq_ complex enlarged (middle) and at the first and last water involved steps with color coded bond lengths (for A) and atoms (for B) are provided above the data panel. The data from other trajectories are provided in Figures S4 and S6.

Figure 2 displays representative snapshots highlighting the successive steps of the halogen exchange reaction observed in one of the three trajectories where the entire reaction takes place. In the other two trajectories, the first step of ICl formation occurs with the generation of OH^–^. Then, OH^–^ starts migrating in the water slab but never reaches to the I side of ICl during our simulation time scale (50 ps). Figure 3 depicts the bond lengths and Hirshfeld partial charges^26^ of the complexes versus the simulation time, where complete halogen exchange reaction occur. Figures S4 and S5 show similar data for other trajectories where entire and partial halogen exchange occur. To explore the conformational space further, we initiated fifteen trajectories from three different geometries with various velocities. (G1-G3, see Methods and Table S1). The bond lengths and Hirshfeld partial charge data along the time from five of those that complete reactive events are shown in Figure S6.

The halogen exchange reaction starts around 5 ps (except those simulations that started from G1-G2 geometries) in all reacting simulations (first vertical black dotted line in Figure 3 and Figures S4–S6). The Cl–O bond of the HOCl molecule breaks and increases >2.5 Å, while the initially formed OH^–^ is released into the water slab (Figure 3A and first columns of Figures S4–S6). When this occurs, Figure 3B and the second columns of Figures S4–S6 show that the partial charge on the I^–^ (purple) ion is transferred to OH (red) of HOCl. At the same time, the I–Cl bond distance becomes ∼2.5 Å upon formation of the ICl molecule. Note that the summation of partial charges of I and Cl charge never reach 0.0 au because part of the electronic charge density is delocalized into the surrounding water molecules. It is important to draw attention to the fact that ICl formation, the first step of the halogen exchange reaction, belongs to a highly important family of reactions in which halogens are formed from HOX and Y^–^. These reactions are by themselves of major atmospheric significance, as mentioned in the introduction.^7,15^ The variation observed here is that ICl is formed at neutral pH, whereas previous reports showed the reactions occurred under acidic conditions.^7^ We also observed the ICl formation in acidic conditions with the addition of protons in two trajectories.The first columns of Figure S7 show the formation of ICl and water after few 100 fs when the I–Cl (blue) bond length becomes ∼2.5 Å and the O–H bond length (light green) becomes ∼1 Å. Cl–O (red) increases >2.5 Å at the same time. Transfer of partial charges from I^–^ (purple) to OH (red) is also indicated by vertical black dotted lines in the second columns of Figure S7. In these cases, no further reaction occurs after HAL[(HOCl)···(I^–^)]_aq_ + H^+^ → ICl + H_2_O.

After formation of ICl in the neutral simulations, the released OH^–^ migrates in water through a Grotthuss-like mechanism.^27−29^ In a few femtoseconds, a nearby water transfers a proton (light green in Figure 3A and Figures S4C, S5A, S5C, S6A, S6G, S6I) to convert the OH^–^ into a water molecule. In other trajectories, either the released OH^–^ shuffles between different water chains and takes the path leading to complete halogen exchange or remains as OH^–^ for long periods (Figures S4A, S6C, S6E). The nascent ICl stays near the surface of water, and its orientation shifts to point the I atom toward a surface water over the range of a few fs to 3 ps. The distance between the O atom of the closest water to the I atom (purple in Figure 3A and first columns of Figures S4–S6) decreases to ∼2.5 Å, indicating the start of HAL[(HOI)···(Cl^–^)]_aq_ complex formation. Interestingly, this change in orientation is not accompanied by an immediate increase in the negative charge on the Cl atom (deep green in Figure 3B and second columns of Figures S4–S6).

**Figure 4:**
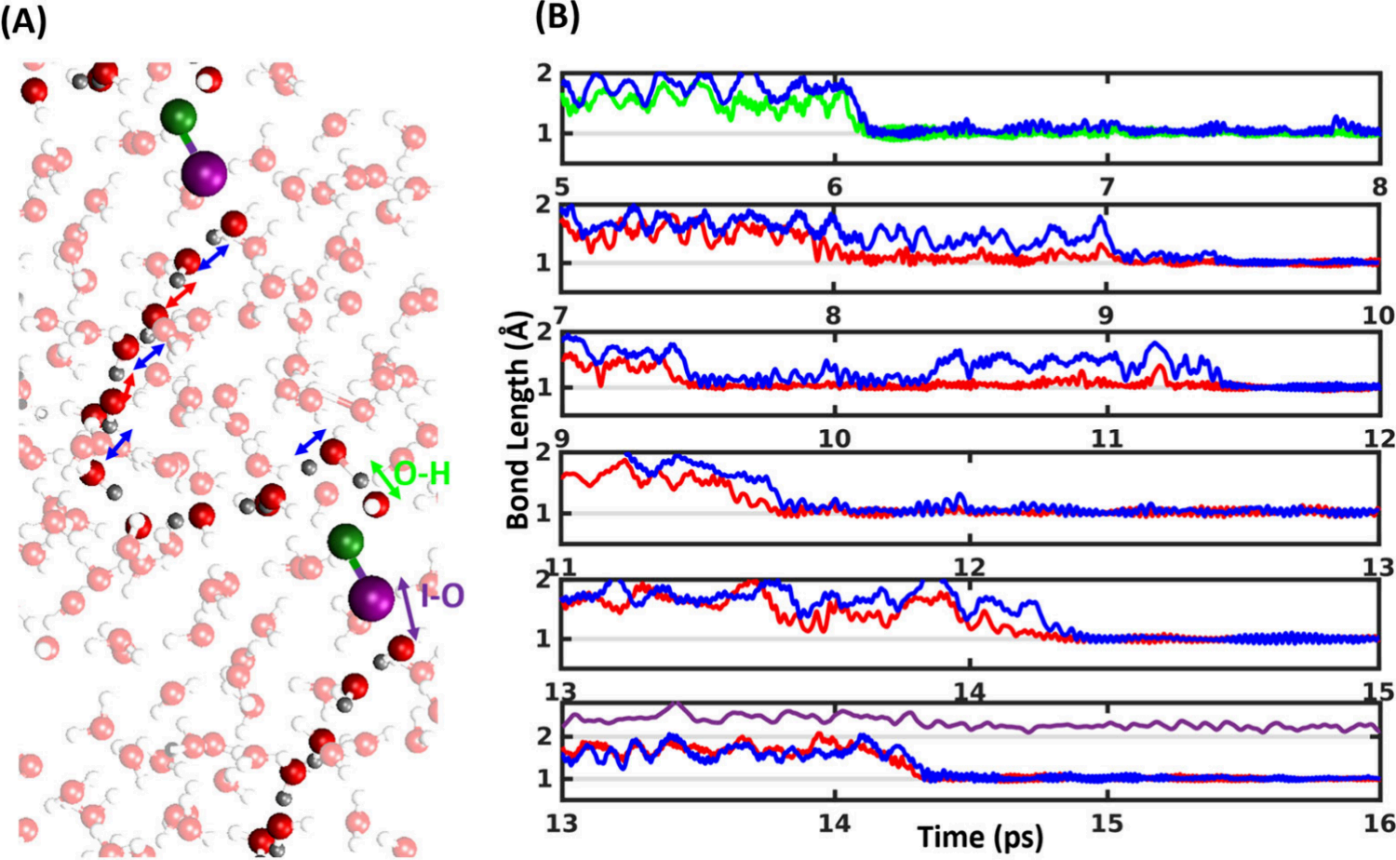
Time evolution of O–H bond lengths depicting OH^**–**^ transport in the halogen exchange reaction. (A) Snapshot at 10 ps showing all the 12 water molecules involved in the Grotthuss-like mechanism step of the complete halogen exchange from HAL[(HOCl)···(I^–^)]_aq_ to HAL[(HOI)···(Cl^–^)]_aq_ in one trajectory. (B) Time evolution of the bond lengths (O–H for different water molecules formed during the Grotthuss-like transport of OH^–^ + H_2_O → H_2_O + OH^–^ and I–O). Different panels in B depict the time required for the formation of water molecules during OH^–^ transport in the halogen exchange reaction. Few color coded bond lengths (for B) are provided in A.

The completion of halogen exchange occurs in eight trajectories among thirty due to the occurrence of three concerted events: (1) the water molecule of the (H_2_O)···(ICl) complex transfers a proton to the solvated hydroxide in the last step of the Grotthuss-like mechanism, (2) the I–Cl bond breaks, and (3) a covalent bond is formed between HO and I, forming the product HOI molecule. Figure 3A and the first columns of Figure S4 and S6 show that the I–O distance (purple) reduces to ∼2.1 Å and the I–Cl bond length increases by ∼0.1 Å. The proton of H_2_O from (H_2_O)···(ICl) shifts to hydroxide ion indicated in the formation of the O–H bond length in the last panel of Figure 4B. Subsequently, the partial charge on Cl also decreases (deep green in Figure 3B and second columns of Figure S4 and S6). These three events result in the formation of the halogen exchange product, the HAL[(HOI)···(Cl^–^)]_aq_. The whole process, from HAL[(HOCl)···(I^–^)]_aq_ to HAL[(HOI)···(Cl^–^)]_aq_, takes ∼10 ps in seven of the trajectories (ranging from 1 to 12 ps, with an average of 4.3 ps, except one trajectory which takes 35 ps) and involves many water molecules ranging from 5 to 12 for the OH^–^ transfer. Figure 4 depicts the O–H bond lengths formed during the OH^–^ transfer process for the trajectory whose bond lengths and partial charge data are presented in Figure 3. This trajectory took ∼8 ps for the whole halogen exchange reaction and involved 12 water molecules. In contrast, where partial reaction occurs, these above three concerted events remain missing. When the halogen exchange reaction proceeds, bond breaking and making do not occur by simple elongation of distance and involve changes of the angles in the broken and formed molecules; simultaneously, the solvation around the complexes also changes. The time evolution of angles broken and formed during HAL[(HOCl)···(I^–^)]_aq_ → HAL[(HOI)···(Cl^–^)]_aq_ and the coordinating atoms around 2.5 Å of I and Cl atoms for one representative trajectory are shown in Figure S8. As angle and coordination number variation along the trajectory provide similar information like the bond length and partial charge variation, for other trajectories we only reported those analyses.

In all cases, however, the chemical participation of water molecules leads to a much faster (by order of magnitudes) halogen exchange than that calculated to occur in the gas phase. The gas-phase halogen exchange mechanism, HAL[(HOCl)···(I^–^)] → HAL[(HOI)···(Cl^–^)], involves three consecutive steps that occur with relatively high barriers of 9.3, 15.5, and 18.4 kcal/mol, yielding unimolecular rearrangement half lifetimes ranging from microseconds to seconds at 300 K (Figure S9). Hence, these simulations demonstrate that water acts as a catalyst accelerating the reaction to completion before 50 ps. In summary, the reaction pathways, intermediate states, and time scales in the gas phase and in the presence of water are even qualitatively different.

Moreover, in three of the remaining simulations, the HAL[(HOCl)···(I^–^)]_aq_ reacts transiently but reverts back to the initial complex (Figure S10). The existence of these transient and partial reacting trajectories could be of interest, as they indicate that modifying reaction conditions might affect reaction yields. In fact, addition of proton mimicking acidic conditions only shows ICl formation (Figure S7).

To summarize, we propose a mechanism for halogen exchange reaction, where water medium participates chemically, resulting in a large catalytic effect.

## Conclusions

We establish the atomistic mechanism of the HOCl + I^–^ → HOI + Cl^–^ reaction at the air–water interface region. The reaction proceeds first by the formation of HAL[(HOCl)···(I^–^)]_aq_ complexes_._ The substantial lifetimes of the halogen-bonded complexes in water at neutral pH suggest that their role as reaction precursors in the chemistry of aqueous environment may be more critical than conventionally assumed. On the other hand, despite the sufficient lifetimes of the HYD[(HOCl)···(I^–^)]_aq_ and HYD[(HOI)···(Cl^–^)]_aq_ complexes in water, those remain unreactive throughout simulations. Our proposed mechanism for the halogen exchange reaction involves three sequential steps: (1) ICl formation starting from HAL[(HOCl)···(I^–^)]_aq_ → ICl + OH^–^, (2) migration of OH^–^ through water molecules by a Grotthuss-like mechanism OH^–^ + H_2_O → H_2_O + OH^–^, and (3) reaction of ICl and OH^–^ (attacking from the I side) to form the HAL[(HOI)···(Cl^–^)]_aq_ complex. The water medium participates chemically in a major way in reactions of the halogen-bonded complexes, qualitatively changing the mechanism, reaction intermediates, and time scales from those of the “dry” isolated complexes in the gas phase. The large catalytic effect of water on the halogen exchange reaction is traced to OH^–^ ion translocation through a water network in a Grotthuss-like process. Thus, simulation of this process requires a sufficiently large slab of water with appropriate boundary conditions to capture the cooperative effect. The water-mediated reaction mechanism outlined in this work should be relevant for other reactions that contain halogens in atmospheric chemistry and more generally in aqueous electrolyte chemistry. Our study suggests the need for future investigations to explore the role of halogen-bonded complexes of Cl^–^, Br^–^, and I^–^ with halogen-containing molecules other than HOCl as precursors or intermediates in their reaction pathways in seawater.

## Methods

### Gas-Phase Rate Calculations

Minimum energy structures of halogen- and hydrogen-bonded complexes of [(HOX)···(Y^–^)] (where X, Y= Cl, I or I, Cl) were adopted from ref (21)(21) calculated at the MP2/aug-cc-pVTZ method/basis set level using Q-Chem software.^30^ For I atoms, the corresponding aug-cc-pVTZ-pp basis set and effective core potential were used.^31^ Geometric parameters of these optimized structures are provided in Table S1. Rate constants were estimated using the Polyrate program^32^ with the Improved Variational Transition State Theory with zero-curvature tunneling (IVTST0) approach.^33^ The necessary barriers and energy differences between products and reactants for these calculations were recalculated by using Gibbs free energies.

### Ab Initio Molecular Dynamics (AIMD) Simulations with Water Slab

We used a water slab model to simulate chemical reactions at the water–air interface region as mentioned in previous publications by our group.^19,34−36^ The unit cell of the water slab is made of 72 water molecules in a 13.47 × 15.56 × 40 Å^3^ rectangular box, where the last side is aligned with the *z*-axis and elongated to contain approximately 15 Å of vacuum on each side of the roughly ∼10 Å thick water slab. Periodic boundary conditions were employed in the *x*- and *y*-directions, forming an infinite surface in the *xy*-plane. A water molecule was replaced from the top layer of the box with the gas-phase optimized [(HOX)···(Y^–^)] species, where X, Y = Cl, I or I, Cl. The system was simulated at 300 K for 15 ps (except reacting ones simulated for 50 ps) using a Nosé–Hoover massive thermostat^37^ with a 0.5 fs time step. This procedure resulted in the formation of the halogen- and hydrogen-bonded [(HOX)···(Y^–^)]_aq_ complexes. The concentration of the complexes in the box is ∼0.8 M; similar concentrations were shown to mimic the halide concentration of seawater and nascent aerosols generated from it.^38,39^ Fifteen trajectories for HAL[(HOCl)···(I^–^)]_aq_ complexes and five trajectories each for HAL[(HOI)···(Cl^–^)]_aq_ and HYD[(HOX)···(Y^–^)]_aq_ complexes were simulated, initializing with different velocities using different SEED parameter in CP2K 7.1.^40^ We have also simulated fifteen trajectories from three different geometries with five different velocities each to expand the conformation sampling. Among these, ten simulations were initiated from geometries where the first step of ICl formation already started (G1 and G2), and the remaining five trajectories from a different geometry (G3). The bond length and the bond angles for the starting geometries are provided in Table S1. Section S1 contains the coordinates for all the initial geometries with water slab used in the AIMD simulations.

All ab initio molecular dynamics simulations were computed using the QUICKSTEP module^41^ of CP2K 7.1^40^ employing the Perdew–Burke–Ernzerhof functional^42^ with a Grimme dispersion correction (PBE-D3).^43,44^ The double-zeta valence polarization basis set (DZVP-MOLOPT-SR)^45^ and the Goedecker-Teter-Hutter (GTH) pseudopotentials^46^ were implemented in the computations. We utilized the Martyna–Tuckerman (MT) algorithm^47^ to treat the long-range electrostatic interactions. The cutoff for the plane-wave basis set is 320 Ry (1 Ry = 1.097 × 10^7^ m^–1^) along with a relative cutoff of 50 Ry. The DFT potential, basis set, and MT algorithm describe well the charge transfer reactions, as shown in the previous studies from our group.^19,34^ All hydrogen atoms were replaced by deuterium to accommodate a larger time step of 0.5 fs for integration to shorten the total simulation time. Multireference methods were not used here, as all atoms and ions involved in the condensed phase reaction are closed-shell species and usage is computationally very expensive. The finite size of our water slab and limited simulation time potentially contribute to trajectories resulting in partial reactions.

In our analysis, we analyzed the Hirshfeld charges^26^ generated from CP2K 7.1.^40^ This charge analysis method involves partitioning the molecule into its constituent atoms and evaluating their deviations from the isolated atoms. Molecular density at each point is distributed according to the contributions of individual atoms to the promolecular density, which is the sum of free contributions from all atoms. This charge analysis is demonstrated to accurately describe the charge transfer reactions in water clusters,^19,48^ providing a well-suited approach for defining similar reactions in our system of interest. We have visualized and analyzed the MD trajectories using the visual molecular dynamics (VMD) application^49^ and tool command language (TCL) routines.

## Supporting Information Available

Additional data showing geometric and electronic properties along trajectories and reaction time parameters, geometric parameters, and Cartesian coordinates (PDF)
