# Supplementary material for: Mechanism of a Halogen Exchange Reaction in Water: Catalysis by Aqueous Media
Source: ACS Cent Sci. 2025 Mar 4;11(4):520–7. doi: 10.1021/acscentsci.4c02228 (PMC12022914; doi:10.1021/acscentsci.4c02228)
Supplement: Supplementary file 1 — oc4c02228_si_001.pdf [file oc4c02228_si_001.pdf]

Supplementary Information

Mechanism of a Halogen Exchange Reaction in Water: Catalysis by Aqueous Media

Imon Mandal,[1#] Itai Zakai,[1#] Natalia V. Karimova,[2] Mark A. Johnson,[3] R. Benny Gerber[1,2]*

[1]The Fritz Haber Center for Molecular Dynamics, Institute of Chemistry, The Hebrew University of Jerusalem, Jerusalem 91904, Israel

[2]Department of Chemistry, University of California, Irvine, California 92697, USA

[3]Sterling Chemistry Laboratory, Department of Chemistry, Yale University, New Haven, Connecticut 06520, USA

***Corresponding Author**

Email for correspondence: robertbenny.gerber@mail.huji.ac.il

[#]These two authors contributed equally to this work

Author ORCIDs

Itai Zakai: 0000-0002-0543-6562

Imon Mandal: 0000-0001-9680-8407

Natalia V. Karimova: 0000-0002-4616-1884

Mark A. Johnson: 0000-0002-1492-6993

R. Benny Gerber: 0000-0001-8468-0258

Table of Contents

Figure S1. RMSD evolution of $[(HOX)\ldots(Y^-)]_{aq}$ (X,Y=Cl,I or I,Cl) complexes…..……….……....3

Figure S2. Time evolution of bond length of HYD$[(HOX)\ldots(Y^-)]_{aq}$ complexes showing solvent separated molecule ion pair formation……………………………………………………………….4

Figure S3. Time evolution of bond lengths of HYD$[(HOX)\ldots(Y^-)]_{aq}$ complexes indicating nonreactive state ……………..………………………………………………………………5

Figure S4. Time evolution of bond lengths and partial charges along trajectories where complete halogen exchange reaction occurs………………………………………………………………6

Figure S5. Time evolution of bond lengths and partial charges along trajectories where partial halogen exchange reaction occurs………………………………………………………………7

Figure S6. Time evolution of bond lengths and partial charges along five trajectories (initiated from geometries other than the gas phase optimized one) where complete halogen exchange reaction occurs……………………………………………………………..………………8-9

Figure S7. Bond lengths and partial charges along two trajectories at acidic pH ………………10

Figure S8. Angles and coordination around ICl along a representative trajectory………………11

Figure S9. Reaction time parameters from rate calculations……………………………………12

Figure S10. Time evolution of bond lengths and partial charges of HAL$[(HOCl)\ldots(I^-)]_{aq}$ complexes showing transient ICl formation…………………………………………………...13

Table S1. Geometric parameters of pre-reactive complexes of $[(HOX)\ldots(Y^-)]$ (X,Y=Cl,I or I,Cl)……………………………………………………………………………………………14

Section S1. Cartesian coordinates of all the geometries used to initiate AIMD simulations……………………………………………………………………………....14-29

Figure S1 RMSD evolution of $[(HOX)…(Y^-)]_{aq}$ (X,Y=Cl,I or I,Cl) complexes

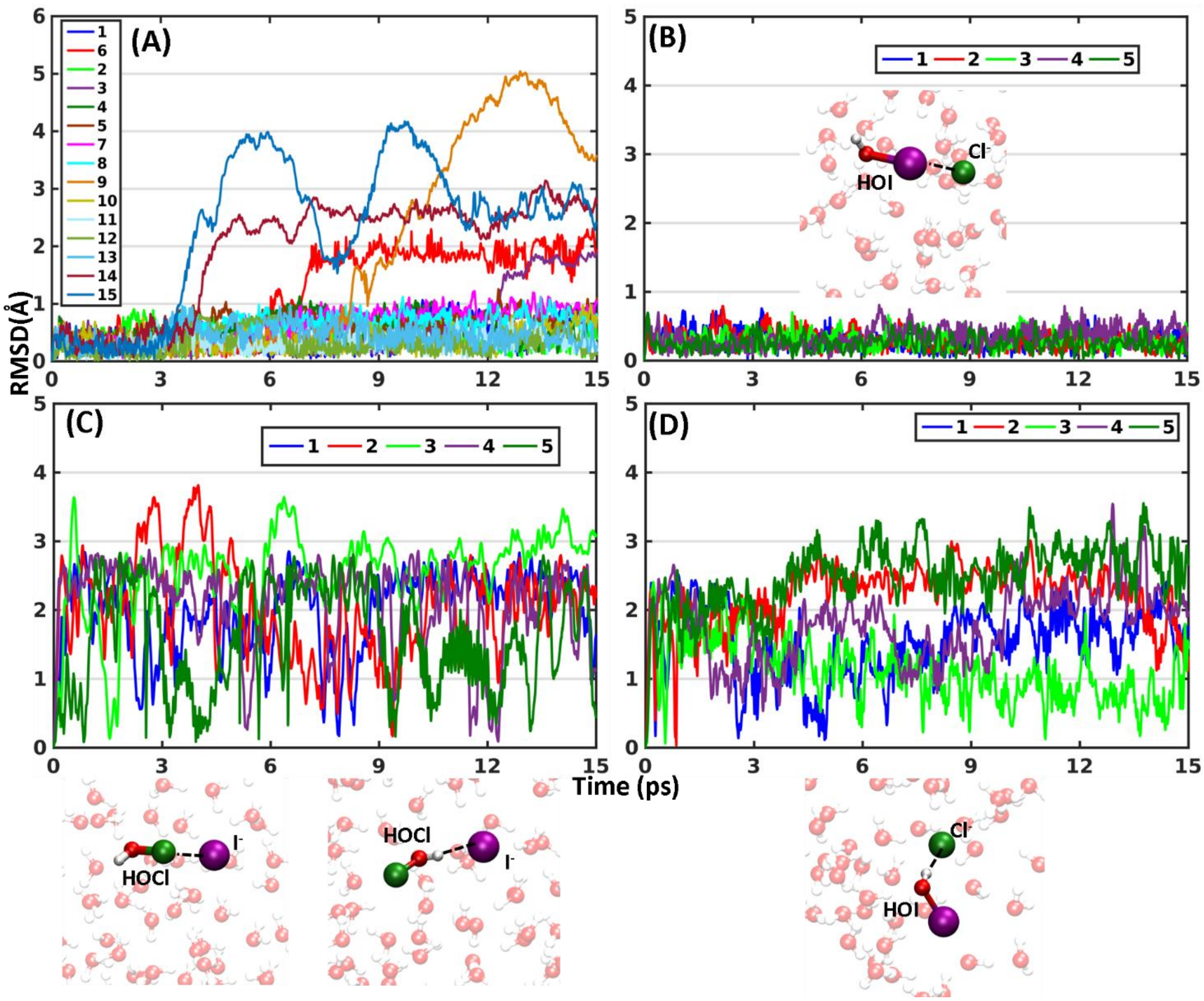

(A) RMSD of the HAL$[(HOCl)…(I^-)]_{aq}$ along the time trajectories for fifteen simulations. The other fifteen trajectories started from geometries other than the gas-phase optimized structure with the water slab have not been shown here as the reference structure has changed. **(B),(C),(D)**: RMSD of the HAL$[(HOI)…(Cl^-)]_{aq}$, HYD$[(HOCl)…(I^-)]_{aq}$ and HYD$[(HOI)…(Cl^-)]_{aq}$ complexes along the time trajectories for all five simulations. Different colors indicate simulations with different velocities (see *Methods*). Snapshots of the stabilized $[(HOX)…(Y^-)]_{aq}$ complexes at time 15 ps for representative simulations are shown in the inset and below the plot.

Figure S2 Time evolution of bond length of HYD[(HOX)…(Y^-)]$_{aq}$ complexes showing solvent separated molecule ion pair formation

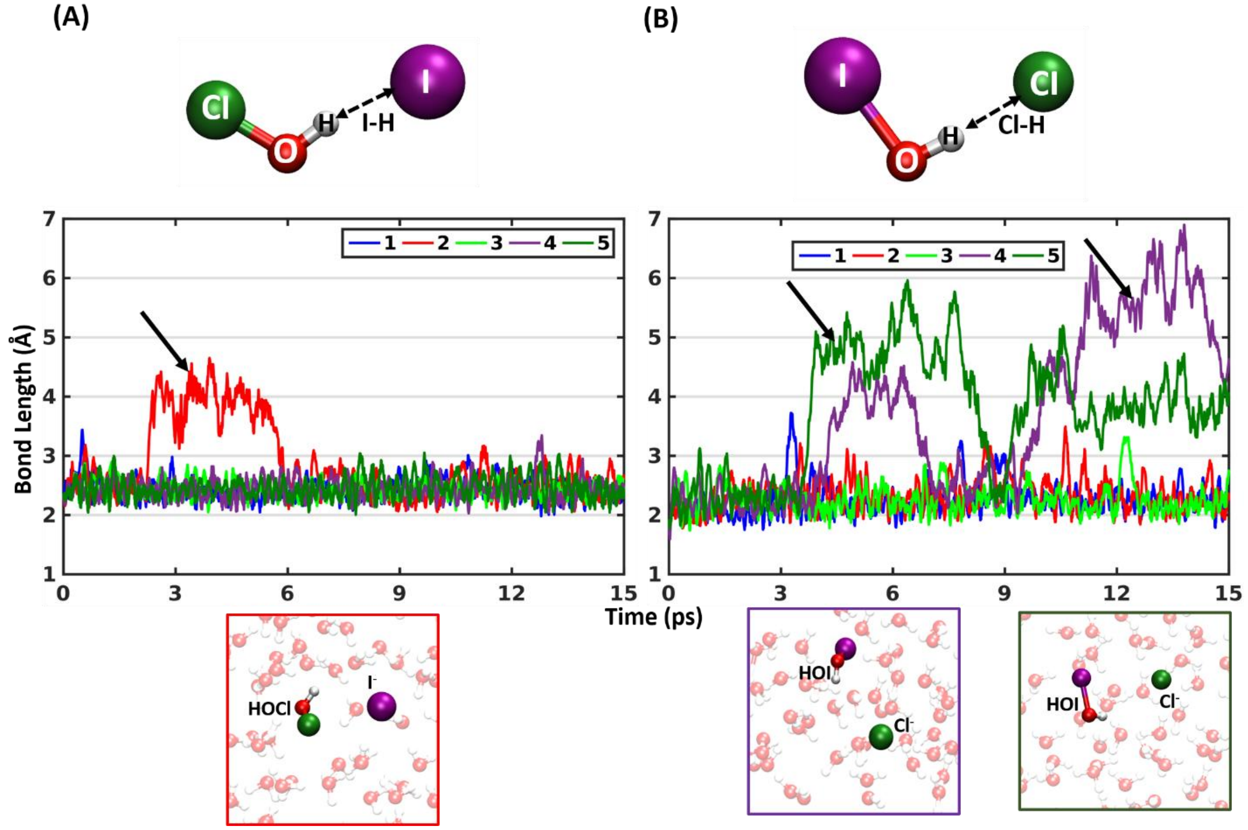

(A) and (B)Y^--H bond lengths of the HYD[(HOCl)…(I^-)]$_{aq}$ and HYD[(HOI)…(Cl^-)]$_{aq}$ along the time trajectories for all five simulations. Different colors indicate simulations with different velocities (see *Methods*). Formation of solvent separated molecular ion pairs indicated by larger bond lengths shown by black arrow. Snapshots of the solvent separated pairs are also provided below with boundary colored same as the trajectory.

Figure S3 Time evolution of bond lengths of HYD[(HOX)…(Y^-)]$_{aq}$ complexes indicating nonreactive state

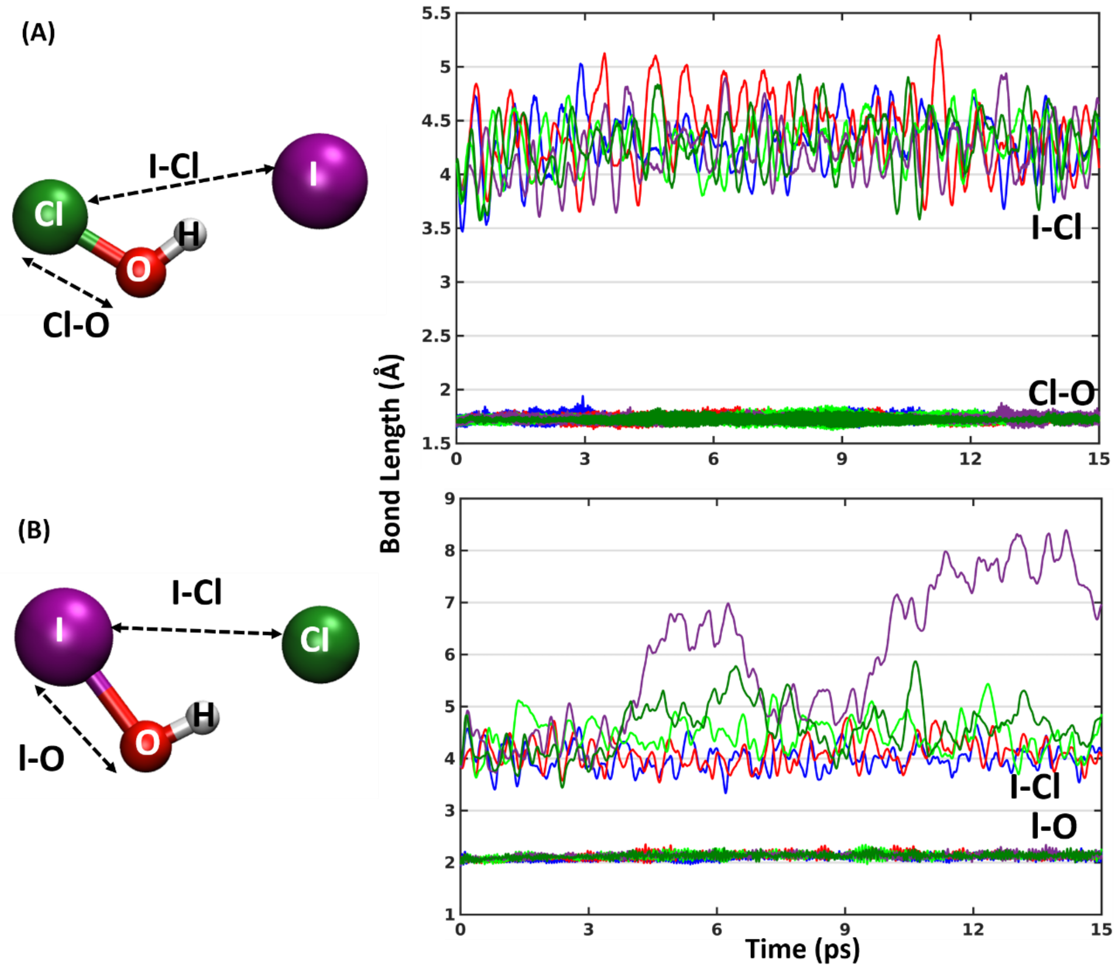

(A) and (B) Bond lengths of the HYD[(HOCl)…(I^-)]$_{aq}$ and HYD[(HOI)…(Cl^-)]$_{aq}$ along the time trajectories indicating no reaction for all five simulations. Different colors indicate simulations with different velocities (see *Methods*).

Figure S4 Time evolution of bond lengths and partial charges along trajectories where complete halogen exchange reaction occurs

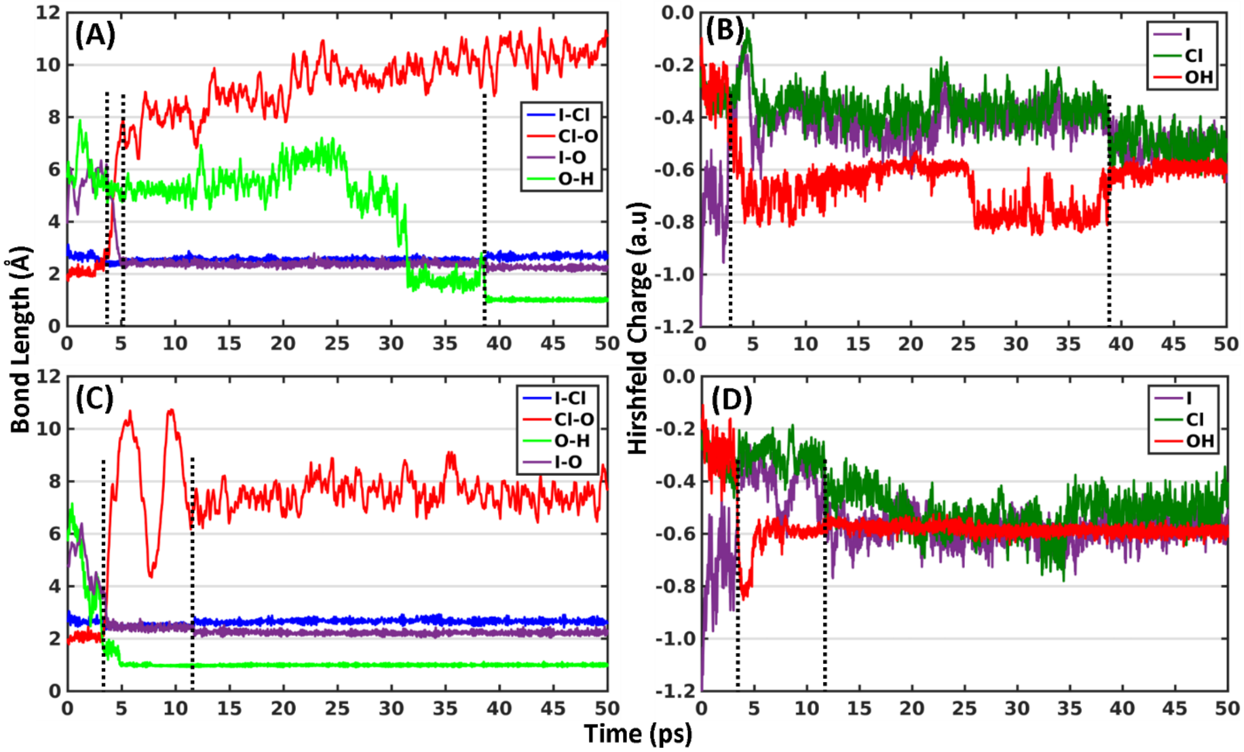

Time evolution of the bond lengths (I-Cl, Cl-O, O-H and I-O) **(A)** and **(C)** and Hirshfeld partial charges (on I, Cl, summation of O and H from HOCl) **(B)** and **(D)** along trajectories where complete halogen exchange occurs. The black dotted lines in all parts are eye guides for the events (mentioned in the text) involved in the halogen exchange reaction. Structure of the HAL[(HOCl)···(I^-)]$_{aq}$ complexes with color coded bond lengths (for **A,C**) and atoms (for **B,D**) are provided above the data panels in Figure 3 of main manuscript where the data for another trajectory have been presented.

Figure S5. Time evolution of bond lengths and partial charges along trajectories where partial halogen exchange reaction occurs

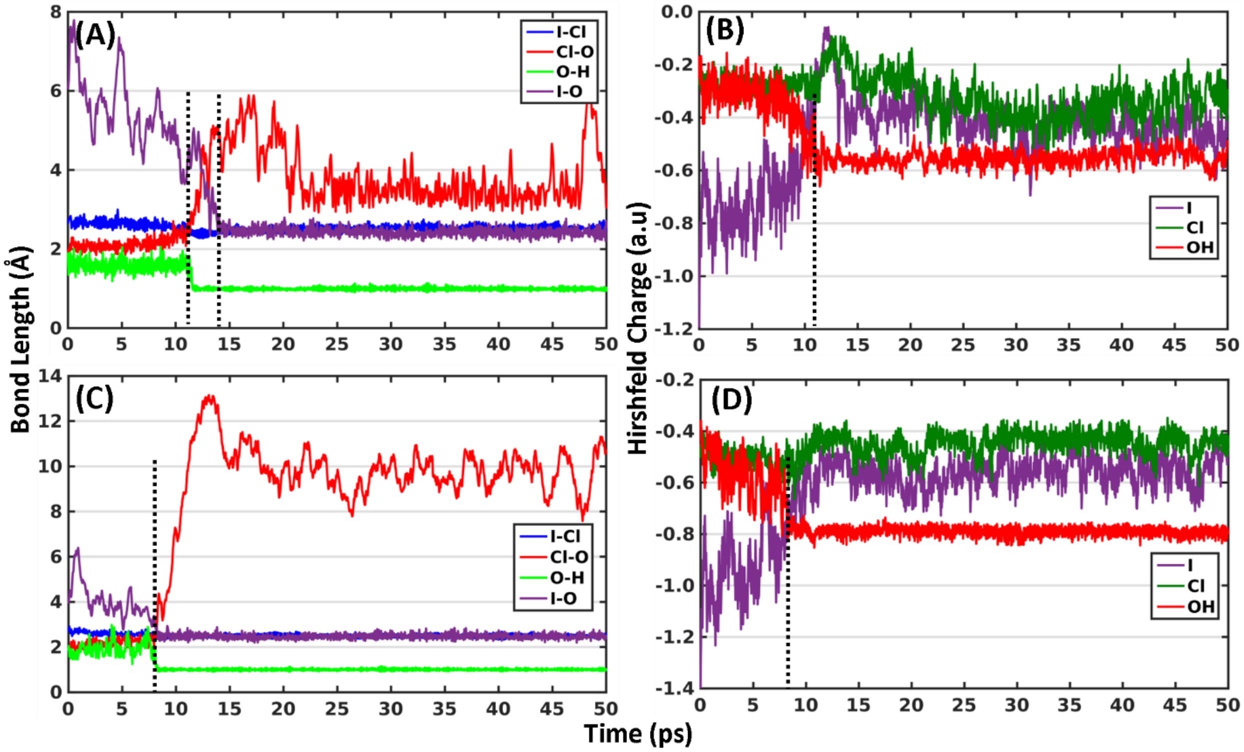

Time evolution of the bond lengths (I-Cl, Cl-O, O-H and I-O) **(A)** and **(C)** Hirshfeld partial charges (on I, Cl, summation of O and H from HOCl) (**B)** and **(D)** along trajectories where partial halogen exchange occurs. The vertical black dotted lines in all parts are eye guides for the events (mentioned in the text) involved in the halogen exchange reaction. Structure of the HAL[(HOCl)···(I^-)]$_{aq}$ complexes with color coded bond lengths (for **A,C**) and atoms (for **B,D**) are provided above the data panels in Figure 3 of main manuscript.

Figure S6 Time evolution of bond lengths and partial charges along five trajectories (initiated from geometries other than the gas-phase optimized one) where complete halogen exchange reaction occurs

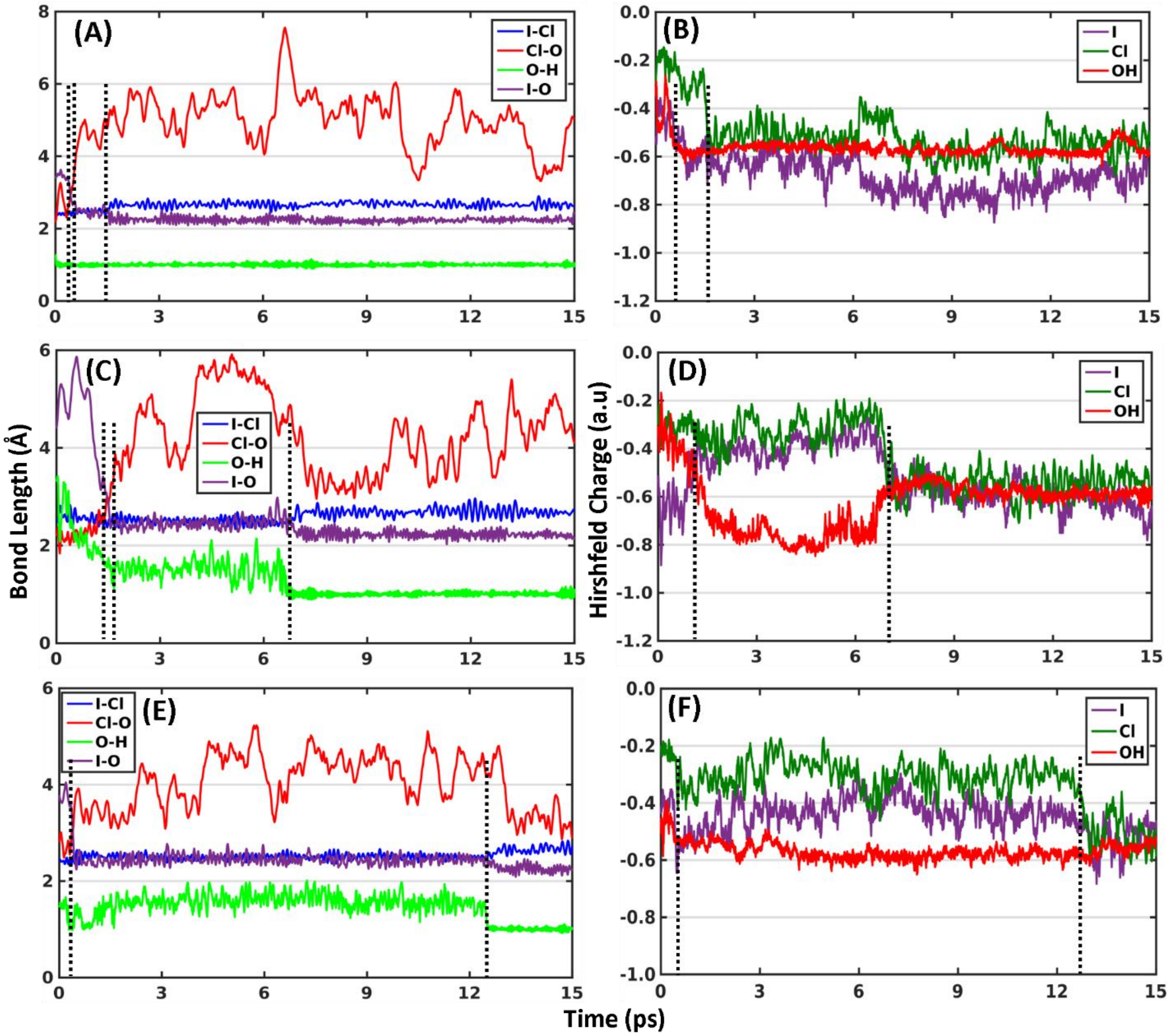

Time evolution of the bond lengths (I-Cl, Cl-O, O-H and I-O) **(A), (C)** and **(E)** and Hirshfeld partial charges (on I, Cl, summation of O and H from HOCl) **(B), (D)** and **(F)** along trajectories where complete halogen exchage occurs. The vertical black dotted lines in all parts are eye guides for the events (mentioned in the text) involved in the halogen exchange reaction. Structure of the HAL[(HOCl)···(I^{-})]$_{aq}$ complexes with color coded bond lengths (for **A,C,E**) and atoms (for **B,D,F**) are provided above the data panels in Figure 3 of main manuscript. The geometric parameters for starting geometry for these simulations are provided in Table S1 (G2 (**A-D)** and G1 (**E-F**)). Plots are continued in the next page.

Figure S6 Time evolution of bond lengths and partial charges along five trajectories (initiated from geometries other than the gas-phase optimized one) where complete halogen exchange reaction occurs

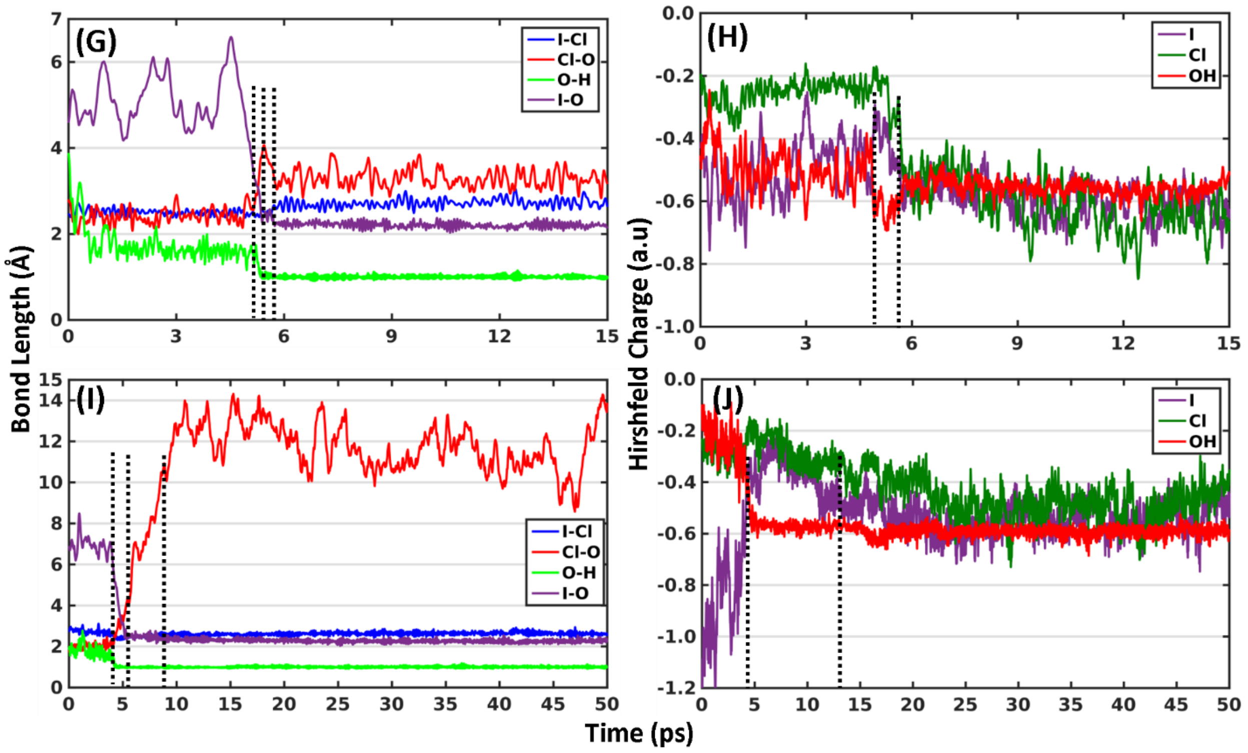

Time evolution of the bond lengths (I-Cl, Cl-O, O-H and I-O) **(G)** and **(I)** and Hirshfeld partial charges (on I, Cl, summation of O and H from HOCl) **(H)** and **(J)** along trajectories where complete halogen exchage occurs. The vertical black dotted lines in all parts are eye guides for the events (mentioned in the text) involved in the halogen exchange reaction. Structure of the HAL[(HOCl)···(I^-)]$_{aq}$ complexes with color coded bond lengths (for **G,I**) and atoms (for **H,J**) are provided above the data panels in Figure 3 of main manuscript. The geometric parameter for starting geometry for this simulation is provided in Table S1 (G1 **(G-H)** and G3 **(I-J)**).

Figure S7 Bond lengths and partial charges along two trajectories at acidic pH

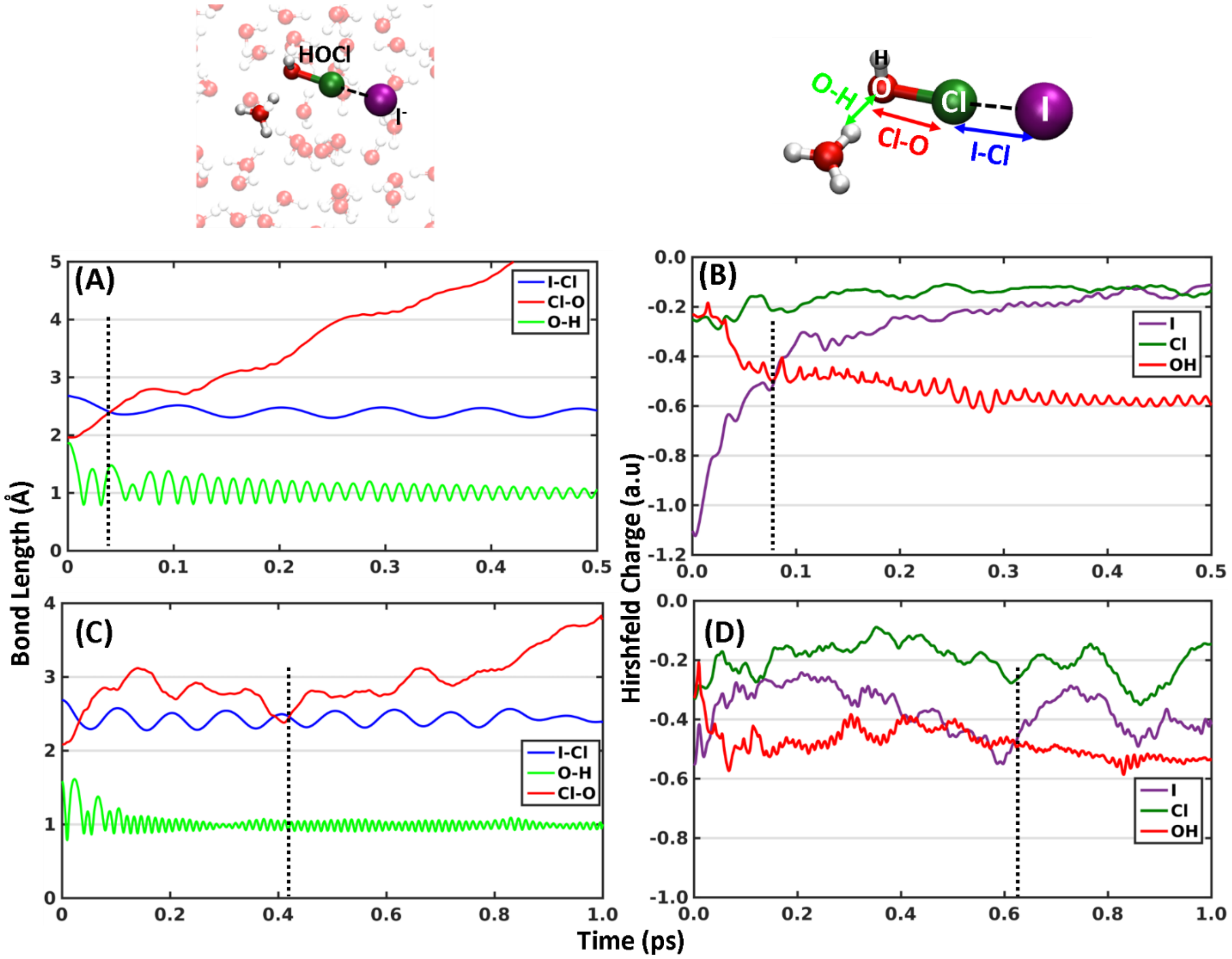

(A) and **(C)** Time evolution of the bond lengths (I-Cl, Cl-O and O-H) and **(B)**and **(D)** Hirshfeld partial charges (on I, Cl and summation of O and H from HOCl) of HAL[(HOCl)···(I^-)]$_{aq}$ complexes along trajectories where proton is added to one neighboring water. The vertical black dotted lines are eye guides for the final time of the non-reversible ICl formation at acidic pH. Although simulations are 15 ps long, data are presented for the first few 100 fs to effectively illustrate the changes in bond lengths and Hirshfeld partial changes during ICl formation. Starting structure of HAL[(HOCl)···(I^-)]$_{aq}$ complexes with the extra proton added on a neighboring water molecule forming hydronium ion from one trajectory and enlarged view of HAL[(HOCl)···(I^-)]$_{aq}$ complex and the hydronium ion with color coded bond lengths (for **A,C**) and atoms (for **B,D**) are provided above the data panels.

Figure S8 Angles and coordination around ICl along a representative trajectory

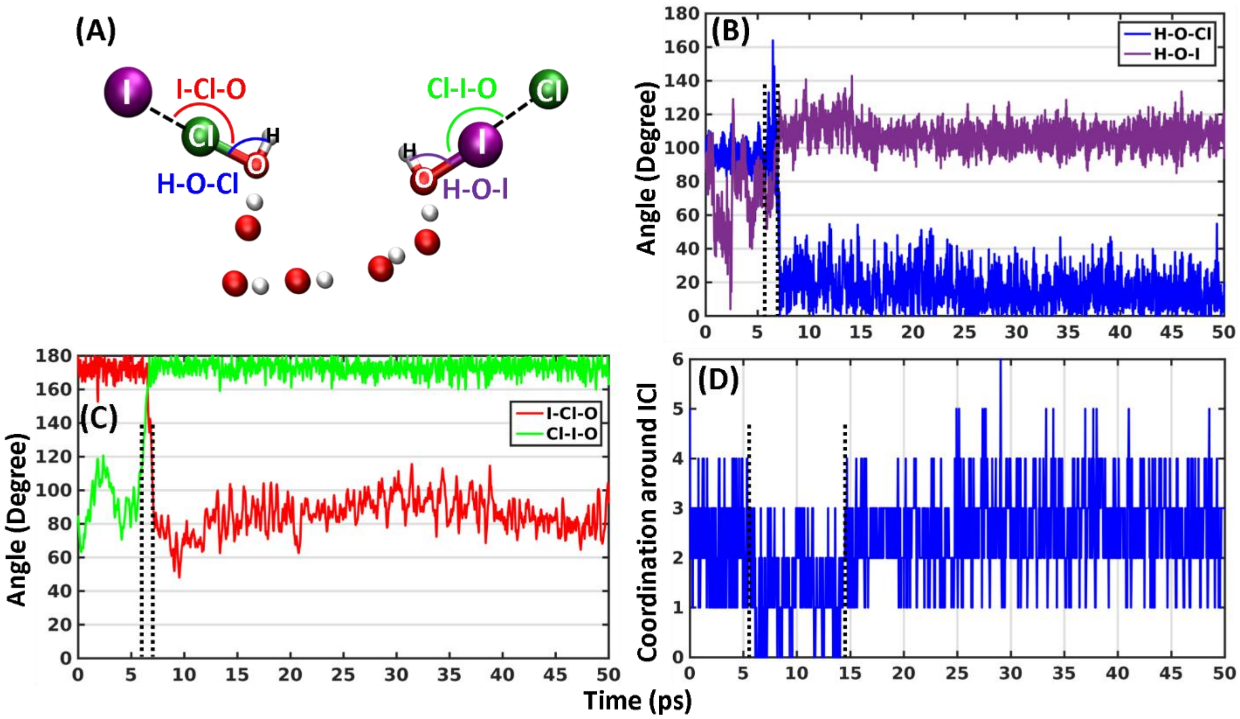

(A) Representative structure of the HAL[(HOCl)···(I^-)]$_{aq}$ and HAL[(HOI)···(Cl^-)]$_{aq}$ complexes connected via a water wire with color coded angles (for **B,C**). **(B)** and **(C)** Time evolution of the four angles broke and formed along a representative trajectory where complete halogen exchange occurs. The black dotted lines in all parts are eye guides for the events (mentioned in the text) involved in the halogen exchange reaction. Bond lengths and Hirshfeld partial charge data for the same trajectory is provided in the Figure 3 of the main manuscript. <H-O-Cl and <I-Cl-O breaks (from the gas phase values) at the same time when the Cl-O starts to increase > 2.5 Å in Figure 3A. Similarly, formation of <H-O-I and <Cl-I-O starts with <H-O-I ~105º and <Cl-I-O >160º when I-O bond decreases to ~2.5Å. **(D)** Time variation of coordinating atoms (including both oxygen and hydrogen atoms) around 2.5 Å of I and Cl atoms. The number decreases when charge transfer occurs from I^- to OH of HOCl as in Figure 3B indicating ICl formation and increases again when partial charge of Cl^- starts decreasing upon completion of HAL[(HOI)…(Cl^-)]$_{aq}$ formation.

Figure S9 Reaction time parameters from rate calculations

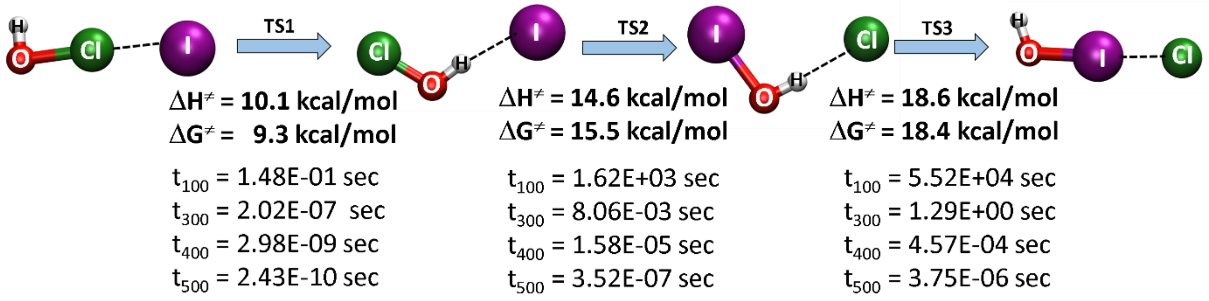

Snapshots and reaction time at 100 – 500 K. Barriers were calculated with enthalpy and Gibbs free energy corrections ($\Delta H^{\neq}$ and $\Delta G^{\neq}$, kcal/mol), method is MP2/aug-cc-pVTZ.

Figure S10 Time evolution of bond lengths and partial charges of HAL[(HOCl)…(I^-)]$_{aq}$ complexes showing transient ICl formation

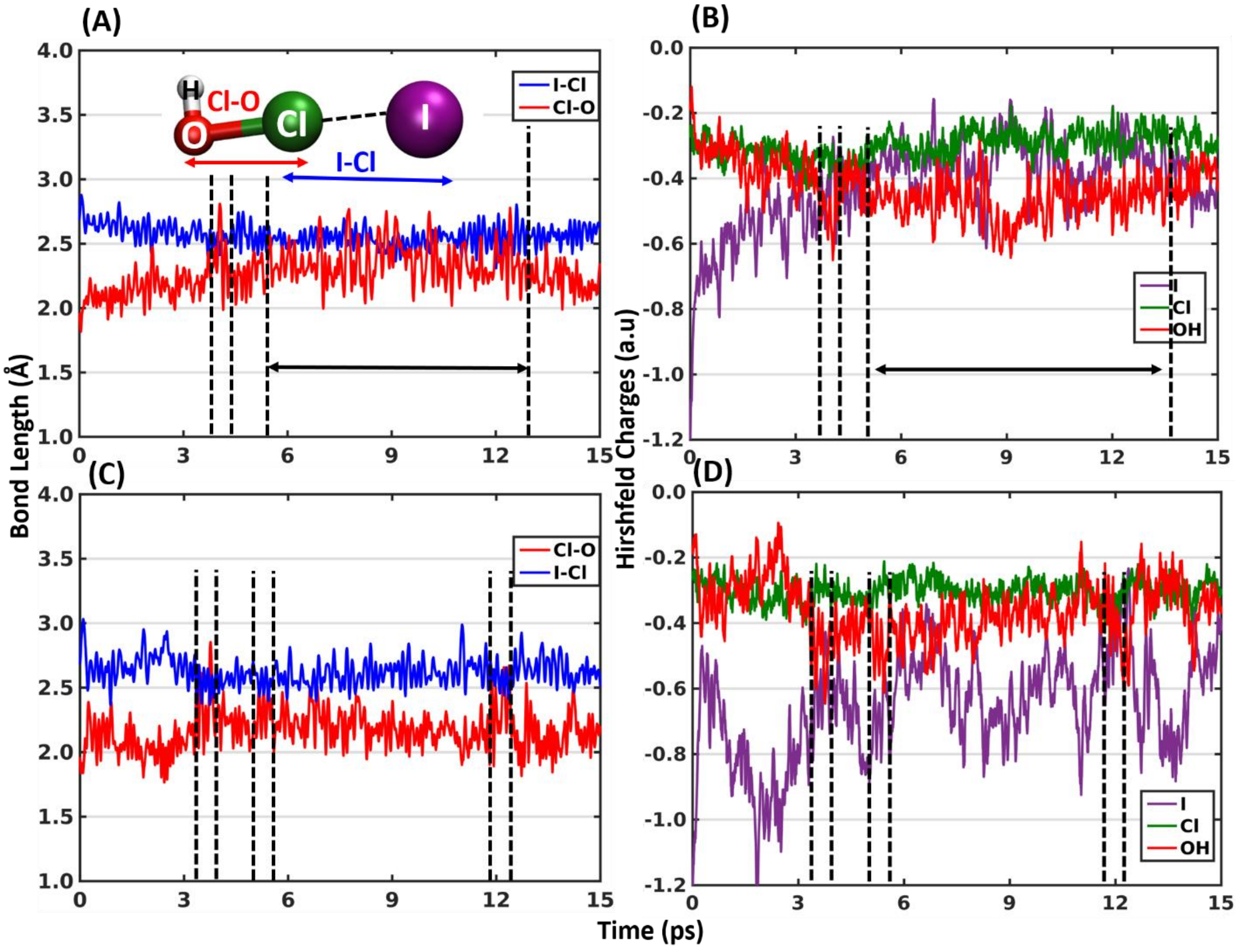

Time evolution of the bond lengths (I−Cl and Cl−O) **(A)** and **(C)** and Hirshfeld partial charges (on I, Cl, and summation of O and H from HOCl) of HAL[(HOCl)···(I^-)]$_{aq}$ complexes along two trajectories where transient ICl formation occurs. The vertical black dotted lines are eye guides for the time of the transient ICl formation at neutral pH. Structure of the HAL[(HOCl)···(I^-)]$_{aq}$ complexes (four atoms H, O, Cl and I) with color coded bond lengths (for A and C) and atoms (for B and D) are provided in the inset of part A. A and C show the I-Cl and Cl-O bond lengths for the trajectories where transient ICl formation happens for few 100s of fs to few ps and subsequently goes back to the initial complex. The I-Cl bond length decreases to ~2.5 Å (blue) and the Cl-O bond length increases (red) at the same time (region indicated with vertical black lines) indicates the ICl formation. The time evolution of Hirshfeld partial charge is consistent with these structural changes (C and D).

Table S1 Geometric parameters of pre-reactive complexes of [(HOX)…(Y⁻)] (X,Y=Cl,I or I,Cl)

Complex Type	Hydrogen-bonded		Halogen-bonded				
Fragment	I^-...HOCl	Cl^-...HOI	HOCl...I^-	HOI...Cl^-	G1	G2	G3
$R_{Y\cdot\cdot H/X}$ (Å)	2.30	1.87	2.68	2.67	2.44	2.47	2.76
$R_{X\text{-}O}$ (Å)	1.69	1.97	1.96	2.12	2.80	2.23	1.78
$R_{O\text{-}H}$ (Å)	1.01	1.03	0.97	0.97	1.03	1.15	1.01
∠HOX	103.29	103.58	95.48	102.25	108.80	79.33	102.24
∠$Y^-\cdot\cdot\cdot$H/X-O	176.14	179.91	179.90	179.18	164.16	170.15	166.18

Gas-phase optimized structure (MP2/aug-cc-pVTZ) of the hydrogen- and halogen- bonded pre-reactive complexes of [(HOX)…(Y^-)] (X,Y=Cl,I or I,Cl) (adopted from Ref 21). *R* is the bond length (Å), and ∠ is the bond angle. Fifth, sixth and seventh column reports the initial geometry of the bare complex for G1, G2 and G3 geometries mentioned in the *Methods* section. Five trajectories were initiated from all the geometries with the water slab expect for the HOCl..I^-, fifteen trajectories were started from this one.

Section S1 Cartesian coordinates of all the geometries used to initiate AIMD simulations

HAL[(HOCl)…(I^-)]$_{aq}$

I	9.189029	8.121552	24.021919
Cl	7.191872	6.625211	23.040710
O	5.732405	5.527859	22.321175
H	5.299266	5.378046	23.175650
O	12.003949	4.418941	15.594741
H	11.716250	4.637028	16.524521
H	11.111578	4.615204	15.152256
O	1.507587	0.464171	21.456297
H	1.528322	1.369797	21.075409
H	1.906476	-0.091955	20.756472
O	4.337439	0.610660	14.975193
H	3.744931	0.872954	14.272036
H	4.712536	1.438476	15.396060
O	12.345058	2.586960	21.276291
H	11.546166	2.685227	20.727768
H	12.100575	2.993004	22.180025
O	5.497654	2.722490	16.409250
H	5.532071	2.541759	17.387110
H	6.446277	2.571913	16.124327
O	2.679368	2.290726	18.626709
H	2.179707	2.647802	19.407707
H	2.714492	2.942420	17.870893
O	3.308627	14.355737	16.608879
H	3.761083	14.910646	15.946655
H	3.950090	13.758657	17.055908
O	3.742844	12.455831	20.613367
H	4.410674	11.014230	20.722525
H	4.028543	13.090712	21.365231
O	10.148878	0.078802	19.212204
H	11.137422	-0.143196	19.210749
H	9.879042	-0.490449	19.983206
O	8.424420	0.063515	17.187994
H	9.083446	0.137020	17.968693
H	7.601868	-0.232337	17.685125
O	5.157011	2.087408	18.968813
H	4.125183	2.093905	18.898510

H	5.410059	1.118910	18.948275
O	11.926863	0.202970	13.686975
H	11.831512	-0.658886	14.096612
H	1.093809	3.304809	14.529705
H	11.752084	0.894825	14.386675
O	2.886671	4.509929	16.762266
H	3.774977	4.223367	16.364243
H	2.207861	4.420211	15.992023
O	1.129653	4.297910	14.725198
H	0.210628	4.447400	15.075416
O	8.114032	11.410165	18.817875
H	8.105985	11.730642	19.739120
H	8.941087	11.692749	18.351843
O	0.776557	12.905968	15.825043
H	1.575561	13.494844	15.707907
H	1.120728	12.378092	16.677109
O	11.565386	13.974611	16.177378
H	12.558624	13.854626	16.143364
H	11.452229	14.824985	16.623116
O	8.899122	0.109970	14.246805
H	9.831272	-0.011714	13.994774
H	8.759865	-0.452877	15.017914
O	5.105784	12.851748	18.285427
H	5.780263	12.069853	18.312872
H	4.489499	12.779109	19.124382
O	1.464071	11.789351	18.161715
H	0.669787	11.831542	18.748987
H	1.851749	10.906376	18.391449
O	6.777704	3.715425	20.341709
H	6.487351	4.454909	20.892166
H	5.967056	3.292462	19.940117
H	2.751237	6.329736	19.435369
O	1.199530	1.073368	14.435840
H	1.295840	0.848261	15.371118
H	0.374487	0.687317	14.168262
O	12.858603	14.773234	19.685669
H	13.607151	14.990648	19.080391
H	12.932794	15.373099	20.437508
O	2.067155	9.530811	26.313026
H	2.657576	10.236409	25.984602
H	1.235623	9.831329	25.891533
O	2.986422	-0.064488	23.740519
H	2.481912	0.077695	22.889309
H	3.537388	0.787255	23.781059
O	9.393322	4.813109	15.014910
H	9.049732	4.005159	15.523276
H	8.982244	5.547926	15.553351
O	9.583688	5.314170	21.153191
H	9.726907	1.746872	19.356113
H	9.769822	6.098742	21.665552
O	4.126707	7.942377	25.289907
H	3.316829	8.329823	25.668503
H	4.045214	8.133842	24.304226

O	7.723634	11.962919	21.571854
H	6.916552	12.394459	21.838478
H	8.478201	9.391760	23.748196
O	6.347180	15.074856	18.923735
H	6.653379	15.119366	19.817524
H	5.884818	14.167410	18.790731
O	10.726364	11.191221	21.432903
H	10.392975	10.229490	21.368380
H	10.684143	11.317350	22.398594
O	9.855590	2.700871	19.549202
H	9.016862	2.891182	19.897007
O	9.653265	14.191776	21.402815
H	10.404632	14.155921	22.030415
H	9.286612	13.254874	21.405075
O	10.616123	11.816242	17.767498
H	11.210422	11.992812	18.513424
H	10.930828	12.454870	17.035101
O	3.451746	6.265915	20.949970
H	4.390543	6.080070	21.064938
H	3.424004	7.190051	21.335888
H	2.779197	12.231120	20.786346
O	6.136966	10.090367	25.498432
H	5.363532	10.558270	25.106010
H	5.754961	9.167987	25.539190
O	4.620347	10.027844	20.670374
H	7.829211	11.335089	22.361465
H	5.527307	9.770836	20.349522
O	13.307140	5.755046	22.552832
H	7.183980	8.347564	19.228125
H	13.031665	5.923781	21.595343
O	7.066858	15.262963	21.710255
H	7.153327	16.228632	21.916481
H	13.090085	6.533101	23.084257
O	11.305552	4.711729	18.213543
H	11.055656	3.868065	18.643572
H	11.985479	5.132381	18.784832
O	9.390586	6.728001	17.517700
H	10.063420	7.386551	17.900381
H	9.863435	5.889731	17.627937
O	8.306973	2.271517	15.786514
H	8.499409	1.557946	16.535572
H	8.401399	1.688309	14.976566
O	11.406178	8.524046	18.800369
H	10.772700	8.752353	19.567928
H	11.643538	9.379865	18.446281
O	13.070812	6.127634	19.682955
H	12.574119	6.915370	19.363672
H	13.958322	6.152160	19.202662
O	3.359921	8.797739	22.565750
H	3.918769	9.285320	21.860146
H	2.375181	8.943588	22.208679
O	9.655396	8.890562	21.046589
H	9.581005	8.274670	21.817686

H	8.713432	8.932951	20.653875
O	12.653072	8.584648	23.391396
H	11.693516	8.566857	23.181219
H	13.103631	8.675440	22.540718
O	-0.503228	1.114147	24.202717
H	0.204389	1.076456	23.508951
H	-0.719969	1.984018	24.510386
O	6.938342	2.233482	22.606611
H	7.651377	2.694191	23.090012
H	6.964593	2.710201	21.694155
O	4.412466	2.117469	23.754120
H	5.304129	2.166646	23.220863
H	4.517103	2.887072	24.326136
O	2.050636	6.382912	18.691456
H	3.194537	9.458901	19.656618
H	2.393290	5.825038	17.950926
O	8.090000	10.314435	23.772995
H	7.378493	10.129132	24.467066
H	11.819025	15.142018	23.900278
O	12.528506	12.175464	19.886555
H	12.560295	13.151169	19.884029
H	11.867277	11.815919	20.562370
O	13.166625	10.604026	25.069387
H	13.515597	11.273716	24.472469
H	12.950496	9.732469	24.490780
O	1.260209	11.909320	21.531134
H	0.470464	12.035105	20.875101
H	1.038178	12.391782	22.414970
O	4.717463	13.770000	22.594519
H	4.276646	14.431834	23.173149
H	5.527401	14.272050	22.287491
O	11.876422	3.816850	23.716249
H	12.396783	4.596274	23.350649
H	10.921824	3.966116	23.845158
O	0.994887	9.299747	21.594833
H	1.264642	9.154741	20.668247
H	1.012825	10.287881	21.647339
O	7.228610	9.163755	19.697449
H	8.882283	5.600714	20.495987
H	7.451939	9.949524	19.082191
O	11.659121	14.245405	23.626554
H	7.968020	14.881942	21.862396
H	11.046687	13.695206	24.200714
O	2.408779	9.209037	19.149580
H	2.570868	8.311316	18.875938
O	0.690831	13.264155	23.604048
H	-0.175873	13.809021	23.725739
H	1.355244	13.837214	24.003334
O	10.201955	11.904243	24.381845
H	9.388964	11.331435	24.352358
H	10.877569	11.295118	24.787104
O	2.334369	4.550501	22.681334
H	1.464975	5.016395	22.773167
H	2.786956	5.148412	22.041264
O	1.361354	2.966828	20.661934
H	0.323720	2.962464	20.769726
H	1.729780	3.485194	21.426622
O	11.332820	1.790814	15.903324
H	10.417972	1.980062	15.593366
H	11.807382	2.650933	15.682632
O	3.646394	11.250874	24.368458
H	3.886485	12.093479	23.938427
H	3.465999	10.578971	23.669903
O	1.587768	-0.249077	18.353443
H	2.131050	-0.739239	17.652411
H	1.793119	0.754721	18.280313
O	8.989500	3.850688	23.368492
H	9.259765	4.225733	22.451008
H	8.610791	4.650224	23.813097
O	4.187022	5.250574	24.575642
H	4.166965	6.193414	24.930584
H	3.315070	5.068305	24.092468
O	7.742130	6.147953	19.418007
H	8.298345	6.582977	18.709190
H	7.394748	5.346527	19.030293

HAL[(HOI)…(Cl^-)]$_{aq}$

Cl	9.500469	8.212456	23.554817
I	7.394643	6.732068	22.863720
O	5.732405	5.527859	22.321175
H	5.301137	5.378693	23.171957
O	12.003949	4.418941	15.594741
H	11.716250	4.637028	16.524521
H	11.111578	4.615204	15.152256
O	1.507587	0.464171	21.456297
H	1.528322	1.369797	21.075409
H	1.906476	-0.091955	20.756472
O	4.337439	0.610660	14.975193
H	3.744931	0.872954	14.272036
H	4.712536	1.438476	15.396060
O	12.345058	2.586960	21.276291
H	11.546166	2.685227	20.727768
H	12.100575	2.993004	22.180025
O	5.497654	2.722490	16.409250
H	5.532071	2.541759	17.387110
H	6.446277	2.571913	16.124327
O	2.679368	2.290726	18.626709
H	2.179707	2.647802	19.407707
H	2.714492	2.942420	17.870893
O	3.308627	14.355737	16.608879
H	3.761083	14.910646	15.946655
H	3.950090	13.758657	17.055908
O	3.742844	12.455831	20.613367
H	4.410674	11.014230	20.722525
H	4.028543	13.090712	21.365231

O	10.148878	0.078802	19.212204
H	11.137422	-0.143196	19.210749
H	9.879042	-0.490449	19.983206
O	8.424420	0.063515	17.187994
H	9.083446	0.137020	17.968693
H	7.601868	-0.232337	17.685125
O	5.157011	2.087408	18.968813
H	4.125183	2.093905	18.898510
H	5.410059	1.118910	18.948275
O	11.926863	0.202970	13.686975
H	11.831512	-0.658886	14.096612
H	1.093809	3.304809	14.529705
H	11.752084	0.894825	14.386675
O	2.886671	4.509929	16.762266
H	3.774977	4.223367	16.364243
H	2.207861	4.420211	15.992023
O	1.129653	4.297910	14.725198
H	0.210628	4.447400	15.075416
O	8.114032	11.410165	18.817875
H	8.105985	11.730642	19.739120
H	8.941087	11.692749	18.351843
O	0.776557	12.905968	15.825043
H	1.575561	13.494844	15.707907
H	1.120728	12.378092	16.677109
O	11.565386	13.974611	16.177378
H	12.558624	13.854626	16.143364
H	11.452229	14.824985	16.623116
O	8.899122	0.109970	14.246805
H	9.831272	-0.011714	13.994774
H	8.759865	-0.452877	15.017914
O	5.105784	12.851748	18.285427
H	5.780263	12.069853	18.312872
H	4.489499	12.779109	19.124382
O	1.464071	11.789351	18.161715
H	0.669787	11.831542	18.748987
H	1.851749	10.906376	18.391449
O	6.777704	3.715425	20.341709
H	6.487351	4.454909	20.892166
H	5.967056	3.292462	19.940117
H	2.751237	6.329736	19.435369
O	1.199530	1.073368	14.435840
H	1.295840	0.848261	15.371118
H	0.374487	0.687317	14.168262
O	12.858603	14.773234	19.685669
H	13.607151	14.990648	19.080391
H	12.932794	15.373099	20.437508
O	2.067155	9.530811	26.313026
H	2.657576	10.236409	25.984602
H	1.235623	9.831329	25.891533
O	2.986422	-0.064488	23.740519
H	2.481912	0.077695	22.889309
H	3.537388	0.787255	23.781059
O	9.393322	4.813109	15.014910
H	9.049732	4.005159	15.523276
H	8.982244	5.547926	15.553351
O	9.583688	5.314170	21.153191
H	9.726907	1.746872	19.356113
H	9.769822	6.098742	21.665552
O	4.126707	7.942377	25.289907
H	3.316829	8.329823	25.668503
H	4.045214	8.133842	24.304226
O	7.723634	11.962919	21.571854
H	6.916552	12.394459	21.838478
H	8.478201	9.391760	23.748196
O	6.347180	15.074856	18.923735
H	6.653379	15.119366	19.817524
H	5.884818	14.167410	18.790731
O	10.726364	11.191221	21.432903
H	10.392975	10.229490	21.368380
H	10.684143	11.317350	22.398594
O	9.855590	2.700871	19.549202
H	9.016862	2.891182	19.897007
O	9.653265	14.191776	21.402815
H	10.404632	14.155921	22.030415
H	9.286612	13.254874	21.405075
O	10.616123	11.816242	17.767498
H	11.210422	11.992812	18.513424
H	10.930828	12.454870	17.035101
O	3.451746	6.265915	20.949970
H	4.390543	6.080070	21.064938
H	3.424004	7.190051	21.335888
H	2.779197	12.231120	20.786346
O	6.136966	10.090367	25.498432
H	5.363532	10.558270	25.106010
H	5.754961	9.167987	25.539190
O	4.620347	10.027844	20.670374
H	7.829211	11.335089	22.361465
H	5.527307	9.770836	20.349522
O	13.307140	5.755046	22.552832
H	7.183980	8.347564	19.228125
H	13.031665	5.923781	21.595343
O	7.066858	15.262963	21.710255
H	7.153327	16.228632	21.916481
H	13.090085	6.533101	23.084257
O	11.305552	4.711729	18.213543
H	11.055656	3.868065	18.643572
H	11.985479	5.132381	18.784832
O	9.390586	6.728001	17.517700
H	10.063420	7.386551	17.900381
H	9.863435	5.889731	17.627937
O	8.306973	2.271517	15.786514
H	8.499409	1.557946	16.535572
H	8.401399	1.688309	14.976566
O	11.406178	8.524046	18.800369
H	10.772700	8.752353	19.567928
H	11.643538	9.379865	18.446281

O	13.070812	6.127634	19.682955
H	12.574119	6.915370	19.363672
H	13.958322	6.152160	19.202662
O	3.359921	8.797739	22.565750
H	3.918769	9.285320	21.860146
H	2.375181	8.943588	22.208679
O	9.655396	8.890562	21.046589
H	9.581005	8.274670	21.817686
H	8.713432	8.932951	20.653875
O	12.653072	8.584648	23.391396
H	11.693516	8.566857	23.181219
H	13.103631	8.675440	22.540718
O	-0.503228	1.114147	24.202717
H	0.204389	1.076456	23.508951
H	-0.719969	1.984018	24.510386
O	6.938342	2.233482	22.606611
H	7.651377	2.694191	23.090012
H	6.964593	2.710201	21.694155
O	4.412466	2.117469	23.754120
H	5.304129	2.166646	23.220863
H	4.517103	2.887072	24.326136
O	2.050636	6.382912	18.691456
H	3.194537	9.458901	19.656618
H	2.393290	5.825038	17.950926
O	8.090000	10.314435	23.772995
H	7.378493	10.129132	24.467066
H	11.819025	15.142018	23.900278
O	12.528506	12.175464	19.886555
H	12.560295	13.151169	19.884029
H	11.867277	11.815919	20.562370
O	13.166625	10.604026	25.069387
H	13.515597	11.273716	24.472469
H	12.950496	9.732469	24.490780
O	1.260209	11.909320	21.531134
H	0.470464	12.035105	20.875101
H	1.038178	12.391782	22.414970
O	4.717463	13.770000	22.594519
H	4.276646	14.431834	23.173149
H	5.527401	14.272050	22.287491
O	11.876422	3.816850	23.716249
H	12.396783	4.596274	23.350649
H	10.921824	3.966116	23.845158
O	0.994887	9.299747	21.594833
H	1.264642	9.154741	20.668247
H	1.012825	10.287881	21.647339
O	7.228610	9.163755	19.697449
H	8.882283	5.600714	20.495987
H	7.451939	9.949524	19.082191
O	11.659121	14.245405	23.626554
H	7.968020	14.881942	21.862396
H	11.046687	13.695206	24.200714
O	2.408779	9.209037	19.149580
H	2.570868	8.311316	18.875938
O	0.690831	13.264155	23.604048
H	-0.175873	13.809021	23.725739
H	1.355244	13.837214	24.003334
O	10.201955	11.904243	24.381845
H	9.388964	11.331435	24.352358
H	10.877569	11.295118	24.787104
O	2.334369	4.550501	22.681334
H	1.464975	5.016395	22.773167
H	2.786956	5.148412	22.041264
O	1.361354	2.966828	20.661934
H	0.323720	2.962464	20.769726
H	1.729780	3.485194	21.426622
O	11.332820	1.790814	15.903324
H	10.417972	1.980062	15.593366
H	11.807382	2.650933	15.682632
O	3.646394	11.250874	24.368458
H	3.886485	12.093479	23.938427
H	3.465999	10.578971	23.669903
O	1.587768	-0.249077	18.353443
H	2.131050	-0.739239	17.652411
H	1.793119	0.754721	18.280313
O	8.989500	3.850688	23.368492
H	9.259765	4.225733	22.451008
H	8.610791	4.650224	23.813097
O	4.187022	5.250574	24.575642
H	4.166965	6.193414	24.930584
H	3.315070	5.068305	24.092468
O	7.742130	6.147953	19.418007
H	8.298345	6.582977	18.709190
H	7.394748	5.346527	19.030293

HYD[(HOCl)…(I^-)]$_{aq}$

I	11.478606	7.868941	24.167711
Cl	8.078174	5.671869	23.475407
O	8.566195	6.466205	24.881405
H	9.444973	6.883290	24.618675
O	-0.190165	3.869724	16.771475
H	0.069045	4.666553	17.217615
H	-1.023595	4.069944	16.217726
O	3.672225	0.498175	22.507565
H	3.994945	1.318925	22.017845
H	3.117835	0.060955	21.875095
O	4.994546	0.547405	15.954775
H	4.840646	1.359445	15.484256
H	5.577225	0.914275	16.703754
O	0.648165	2.083845	21.530827
H	0.312085	2.567085	20.777895
H	0.422656	2.563195	22.407715
O	6.958725	4.373595	17.039455
H	6.713205	4.001685	17.869366
H	7.710916	3.815465	16.707766
O	3.805805	2.711435	18.158375

H	3.390185	2.859325	19.064405
H	3.500775	3.466425	17.595585
O	4.288905	13.627095	16.867954
H	4.808235	14.213595	16.325054
H	4.916025	13.634936	17.649775
O	4.925495	13.041105	20.927555
H	5.784405	11.325895	20.310905
H	5.247205	13.552665	21.721785
O	11.383535	0.679425	19.949915
H	12.241075	0.733905	19.446165
H	11.493715	0.027495	20.635645
O	9.492655	2.340965	19.617245
H	10.299425	1.738475	19.734055
H	8.727165	1.797185	19.934685
O	6.107475	3.394135	19.292934
H	5.237325	3.226275	18.830585
H	6.373995	2.582975	19.790855
O	12.775515	0.648865	13.799185
H	12.935064	-0.101545	14.423446
H	2.145485	3.165705	15.524305
H	12.242805	1.241485	14.250936
O	5.005335	6.052205	16.075766
H	5.721156	5.317125	16.202005
H	4.211175	5.445585	15.920735
O	2.344605	4.107315	15.897206
H	1.458615	4.390755	16.262375
O	8.735835	12.795825	19.037716
H	8.868956	12.572975	20.009315
H	9.588105	12.877355	18.608475
O	1.687845	12.669435	16.416586
H	2.628045	12.886946	16.558495
H	1.521945	11.942725	17.125515
O	13.189375	14.608145	16.036295
H	13.877246	13.896296	16.249855
H	13.181905	15.204295	16.776655
O	8.707174	15.417135	17.050764
H	9.492344	14.793175	17.241236
H	7.895175	14.903975	17.198814
O	6.136655	14.153476	18.917585
H	6.877045	13.523496	19.110134
H	5.438185	13.846936	19.655245
O	0.831565	11.215815	18.433846
H	0.498396	11.927996	19.037754
H	1.630975	10.890046	18.884504
O	7.465735	5.657395	19.912865
H	7.537475	5.362195	20.852535
H	6.877175	4.983375	19.603895
H	3.870165	7.079575	18.966576
O	1.989175	1.480706	14.914226
H	1.957815	1.034025	15.842605
H	1.107985	1.342455	14.463825
O	1.657815	15.071725	20.482494
H	2.209726	15.421245	19.743885
H	1.234086	15.828484	20.855158
O	3.748085	9.748546	26.200817
H	3.964735	10.665806	25.879595
H	2.750635	9.777305	26.040174
O	4.203715	1.078255	25.657097
H	4.054286	1.089005	24.692795
H	4.849435	1.830625	25.816376
O	11.102685	3.728435	15.015775
H	10.370685	3.287715	15.577725
H	10.730494	4.576445	14.763025
O	11.176885	7.153215	20.526705
H	11.711515	2.793165	21.494347
H	10.945595	6.700145	21.353495
O	5.359685	8.355375	24.546875
H	4.815175	8.677345	25.411156
H	4.832275	8.991395	23.993685
O	9.619855	12.077775	21.624735
H	9.568695	11.724766	22.562666
H	10.367216	10.020735	24.083395
O	6.987226	1.137795	20.371334
H	7.292126	0.926174	21.368505
H	6.596395	0.354375	19.967125
O	12.806165	11.494096	22.163418
H	12.277115	10.739635	21.826984
H	12.558155	11.529845	23.116245
O	11.184835	3.632195	21.412827
H	10.459215	3.324375	20.843197
O	10.422905	14.825955	22.333565
H	10.869716	14.028186	22.683306
H	9.995875	14.412705	21.545685
O	11.256455	13.872305	17.998236
H	12.016755	13.358346	18.340405
H	11.623695	14.160505	17.118885
O	5.188625	7.220005	20.384714
H	6.164765	6.913805	20.403795
H	5.046785	8.257625	20.481966
H	3.930355	12.915045	21.090315
O	7.181695	10.371375	25.420055
H	6.560415	11.076675	25.227726
H	6.800086	9.552106	24.979095
O	5.705025	10.524735	19.775894
H	10.640725	11.822285	21.512606
H	6.591895	10.088855	19.619345
O	1.027395	5.229645	22.513374
H	8.565435	8.912025	18.690035
H	0.743986	5.169445	21.546017
O	7.871965	0.580385	22.757507
H	8.158555	1.403475	23.308495
H	1.159246	6.159215	22.700897
O	13.591296	4.556185	20.004055
H	12.734216	4.719955	20.406807
H	13.705045	5.332105	19.351154
O	11.061505	7.075095	16.517336

H	11.779655	7.733345	16.715824
H	11.501695	6.223195	16.792564
O	9.229585	2.774925	17.029474
H	9.431335	2.623935	17.999905
H	9.125245	1.827985	16.726034
O	12.929985	8.783645	17.925655
H	12.473155	9.009505	18.805525
H	13.527245	9.668145	17.875635
O	0.487016	6.269475	17.980505
H	0.233175	7.227875	17.919134
H	1.513346	6.243665	18.160404
O	4.644676	10.141175	22.526005
H	4.746215	10.524075	21.617214
H	3.673476	9.971455	22.555367
O	11.319295	9.834405	20.238626
H	11.412735	8.922305	20.703056
H	10.368885	9.873805	19.899084
O	1.059365	7.965755	23.260654
H	0.070526	8.079145	23.142614
H	1.495405	8.414135	22.518805
O	1.324895	1.291285	25.729406
H	2.341575	1.361535	25.715324
H	0.981745	2.008915	25.171385
O	8.512275	2.822925	24.064434
H	9.399235	2.975255	24.385508
H	8.363076	3.586135	23.499697
O	5.918715	3.207565	25.617905
H	6.760965	2.880455	25.231827
H	5.696685	3.987465	25.163628
O	3.394235	7.041335	18.101246
H	4.240265	10.001945	18.891926
H	4.024355	6.726485	17.428156
O	9.894725	10.897555	24.184875
H	9.072835	10.687415	24.789305
H	14.350795	15.287636	24.887245
O	13.198935	13.006645	19.970675
H	13.869155	13.783135	19.954926
H	13.127345	12.550825	20.900227
O	1.013696	10.053655	25.321825
H	1.496936	10.695465	24.750927
H	0.889225	9.271595	24.689167
O	2.511235	12.465745	21.691666
H	2.107386	13.337395	21.450165
H	2.441516	12.395185	22.763775
O	5.679055	14.323485	23.174847
H	4.900055	14.857595	23.027584
H	6.460166	14.952565	23.119427
O	13.762446	3.081185	23.902836
H	14.230985	3.958545	23.726976
H	12.869555	3.413955	24.225655
O	1.885005	9.565945	21.221836
H	2.347696	9.546965	20.340244
H	1.637486	10.488906	21.345104
O	8.139746	9.753695	18.976015
H	10.286045	7.163725	20.107277
H	8.232495	10.405265	18.268536
O	14.181635	14.477865	24.363544
H	8.643135	0.117254	22.491415
H	13.377375	14.165565	24.830547
O	3.281485	9.755685	18.798056
H	3.225885	8.884675	18.338844
O	2.206415	12.224745	24.276407
H	1.807135	13.043465	24.595964
H	3.158905	12.288915	24.653717
O	12.001686	12.007795	24.791155
H	11.059325	11.707495	24.798185
H	12.529185	11.341395	25.343445
O	3.915797	5.216895	21.771435
H	2.948526	5.413265	21.898706
H	4.337785	5.882385	21.229155
O	3.192345	2.747165	20.717295
H	2.305655	2.581845	21.110575
H	3.569625	3.586895	21.071136
O	13.278255	1.330005	18.031555
H	14.165245	0.902195	17.983295
H	13.240115	2.169705	17.561575
O	5.014215	12.001905	24.844416
H	5.277955	12.957695	24.554737
H	5.216485	11.461915	24.058416
O	2.282545	0.184025	17.761715
H	2.788385	-0.507725	17.259645
H	2.959876	0.883685	17.988415
O	11.209745	3.671416	24.315004
H	11.033935	3.909223	23.341644
H	10.943915	4.459693	24.790344
O	5.361866	5.671215	24.252575
H	5.143137	6.641775	24.397057
H	4.858306	5.407815	23.430275
O	9.229305	7.400795	18.450705
H	9.836605	7.327635	17.651415
H	8.624536	6.635845	18.449816

HYD[(HOI)…(Cl^-)]$_{aq}$

Cl	9.985826	8.158057	22.625462
I	6.596109	6.300089	22.777147
O	7.792525	7.029775	24.154970
H	8.570747	7.428965	23.612446
O	12.570695	2.587894	14.521221
H	12.745114	3.076445	15.372430
H	11.889185	3.226765	14.112061
O	1.820306	0.274725	22.112301
H	1.674356	1.080665	21.488621
H	1.013786	-0.176605	21.801279
O	5.645005	-0.569765	15.258550
H	4.699346	-0.563465	14.931411

H	5.791875	0.396415	15.491951
O	12.512436	2.496395	19.540600
H	12.487335	2.948955	18.648149
H	11.903375	3.052525	20.067341
O	5.893855	2.253465	16.064369
H	5.988316	2.439695	17.055651
H	6.718505	2.636945	15.660300
O	3.083095	1.700705	18.710340
H	2.652755	2.150385	19.466181
H	2.667925	2.108515	17.904989
O	5.642766	14.154325	17.781111
H	5.573875	14.382195	16.737040
H	5.930675	13.233805	17.827749
O	5.087405	11.951035	20.872660
H	6.030655	9.807595	18.009920
H	5.606105	12.580675	21.378500
O	9.722655	13.773305	18.580469
H	10.267755	13.331686	17.878401
H	10.296705	14.061525	19.306709
O	9.217475	0.704125	16.969860
H	9.802855	-0.063965	17.136629
H	8.475135	0.546325	17.636410
O	5.815556	2.606025	18.615841
H	4.879945	2.264195	18.640619
H	6.406216	1.740085	18.722820
O	11.254945	12.518486	16.632099
H	12.118305	12.026826	16.677799
H	2.183695	1.849505	13.770100
H	11.237325	12.862596	15.739780
O	3.043645	2.805175	16.337460
H	4.056146	2.682065	16.281719
H	2.709845	2.887175	15.418681
O	1.956675	2.784145	13.713311
H	0.996675	2.977295	13.806530
O	8.859055	11.220525	19.663040
H	8.561505	11.323905	20.651991
H	9.087855	12.156735	19.409611
O	2.291936	10.480395	16.432360
H	3.134555	10.720975	15.966820
H	2.484177	9.564375	16.833561
O	14.022035	14.070476	14.127740
H	14.158545	13.193436	14.592010
H	13.179515	14.404726	14.520870
O	8.538224	0.663295	14.299440
H	8.890065	0.503995	15.195250
H	7.660214	0.089825	14.382371
O	6.117055	11.541096	18.487730
H	7.082695	11.455116	18.683050
H	5.711775	11.612665	19.436440
O	13.293665	10.849035	17.104410
H	13.606616	11.078236	18.066469
H	14.266846	10.871546	16.690750
O	7.013606	3.928505	20.619440

H	6.638915	4.787565	20.897240
H	6.372445	3.524595	19.966999
H	3.227375	5.118155	18.425591
O	2.817895	15.640155	14.699701
H	2.806605	16.096195	15.546570
H	1.964215	15.163635	14.570040
O	12.908925	15.374105	20.360142
H	13.243695	14.920185	19.539780
H	12.701735	16.248276	20.025949
O	2.457565	9.797445	26.095901
H	3.059905	10.513585	26.361483
H	1.573495	10.136965	25.748842
O	4.070815	-0.231545	23.547371
H	3.322265	0.008985	22.956829
H	4.355045	0.692645	23.813961
O	10.751084	4.434345	13.638520
H	9.829644	4.055155	13.811970
H	10.770745	5.317965	14.065490
O	11.136955	4.209575	21.231730
H	9.778085	2.409895	17.248940
H	11.869535	4.838475	21.337221
O	4.414665	8.910005	24.343069
H	3.486705	9.100015	24.645340
H	4.418575	9.123156	23.374090
O	8.147945	11.451545	22.218981
H	8.677055	10.776065	22.722759
H	9.201976	9.412115	25.085699
O	7.267605	16.017534	18.836861
H	7.756715	15.833486	19.736179
H	6.663295	15.236206	18.436081
O	11.412195	11.917035	21.840282
H	11.402585	11.865905	22.840750
H	10.632495	11.341775	21.575430
O	9.853235	3.425705	17.266270
H	9.709985	3.897815	18.168690
O	10.484756	14.334656	21.298380
H	11.257555	14.728505	20.862263
H	10.993466	13.465136	21.589729
O	0.632185	14.187925	18.201290
H	1.599566	14.382086	18.363621
H	0.338246	13.343256	18.515120
O	3.774055	5.741095	20.102560
H	4.629655	5.751625	20.562389
H	3.541135	6.751655	20.083042
H	4.118365	12.064895	20.927410
O	6.537405	10.359886	25.572760
H	6.090165	11.161065	25.762711
H	5.988955	9.768835	25.006941
O	5.835145	8.832545	18.046329
H	7.767585	12.184345	22.802191
H	6.507646	8.316335	17.561720
O	13.169925	6.008805	20.971741
H	9.016055	7.047435	17.022240

H	13.334815	6.101875	19.966480
O	8.222485	15.569566	21.388910
H	8.274435	16.335754	22.050831
H	13.139715	6.945525	21.250919
O	12.668045	3.840035	17.130640
H	11.666015	3.771475	16.953560
H	12.873695	4.814845	17.387659
O	10.980405	6.600055	15.590260
H	11.539456	7.270735	16.049339
H	11.343135	5.799545	16.096060
O	8.287265	3.356715	14.959210
H	8.862665	3.456965	15.762010
H	8.400295	2.416825	14.609221
O	12.424045	8.195465	17.047689
H	11.830965	8.450955	17.794710
H	12.821495	9.092606	16.767570
O	0.145626	6.233945	18.225229
H	0.160716	7.063745	17.627581
H	1.118905	5.999935	18.095289
O	5.040966	9.380185	21.640900
H	5.812187	8.764905	21.653130
H	5.351915	10.281845	21.435602
O	10.081465	8.867555	18.985130
H	10.357445	8.483435	19.794550
H	9.651175	9.712295	19.170170
O	0.163825	8.980915	20.960873
H	0.014596	9.863965	20.505732
H	1.096816	8.819755	20.823191
O	0.721605	0.398085	24.703920
H	1.065945	0.694085	23.843281
H	-0.264545	0.587175	24.601669
O	7.782526	2.317285	22.551081
H	8.546355	2.921395	22.809999
H	7.381075	2.959605	21.891319
O	5.065145	2.277655	23.544331
H	6.059145	2.385055	23.476370
H	4.654495	3.148325	23.579710
O	2.780145	5.286535	17.591431
H	4.277915	8.191075	17.466021
H	2.677185	4.514255	17.070860
O	9.349985	10.222865	25.594002
H	8.524295	10.744265	25.545971
H	14.482766	14.232435	25.272282
O	13.713095	11.576035	19.684561
H	14.505165	12.067745	20.032990
H	12.975916	11.745625	20.332460
O	13.768305	10.128446	23.534430
H	14.446746	10.828205	23.298170
H	13.421906	9.757885	22.690102
O	2.329456	12.833755	20.782909
H	2.521066	13.682246	20.311981
H	2.075216	13.049145	21.711720
O	6.363165	13.957995	22.641470
H	5.727295	14.637465	22.917002
H	6.985895	14.481855	22.101210
O	11.098455	0.965205	23.950520
H	10.564335	1.741305	24.157591
H	10.630846	0.578315	23.190069
O	3.069356	8.368245	20.258120
H	2.981656	8.494935	19.282070
H	3.916826	8.802746	20.604919
O	8.516305	6.932925	17.831560
H	10.400315	4.439675	20.560320
H	8.957115	7.684465	18.371250
O	14.181086	13.315465	25.538910
H	9.209695	15.180645	21.320801
H	13.275075	13.167136	25.149750
O	3.308415	8.048025	17.341591
H	3.107105	7.063875	17.450890
O	1.834825	12.276735	23.285191
H	1.371535	12.882375	23.964741
H	2.795615	12.287235	23.613400
O	11.712205	11.558735	24.523352
H	10.995036	11.120395	25.023191
H	12.290405	10.775255	24.215031
O	2.012316	4.949315	22.067659
H	1.150846	5.302985	21.730539
H	2.630965	5.442525	21.479580
O	1.816955	2.638315	20.584782
H	0.847155	2.771955	20.380180
H	2.090175	3.462045	21.132010
O	11.897446	-0.156235	15.647711
H	12.426936	-0.092355	16.443991
H	11.915605	0.734725	15.160480
O	4.167255	12.809705	24.551571
H	4.332025	13.770716	24.287609
H	4.791935	12.250115	24.066441
O	3.073736	-0.894845	18.915150
H	4.050556	-1.169275	18.596821
H	3.183685	0.083075	18.834860
O	9.807265	3.789006	23.443720
H	10.475215	4.046674	22.744011
H	9.511985	4.707565	23.732059
O	3.928725	6.273565	23.817480
H	3.899266	7.080425	24.316811
H	3.007066	5.980555	23.758991
O	9.360005	4.824485	19.410070
H	9.247206	5.741505	19.002701
H	8.508206	4.523445	19.804640

G1 (with water slab)

I	10.335909	8.994499	23.502399
Cl	8.867493	7.045474	23.596104
O	7.855909	4.434181	23.756809
H	8.619627	3.798800	24.016140

O	11.730235	2.841498	18.329779
H	11.558436	2.005716	18.836481
H	11.067482	3.033773	17.597446
O	3.591652	0.806324	22.709911
H	3.906726	1.616302	22.233580
H	3.235396	0.367889	21.869375
O	6.357988	1.000591	15.620550
H	5.485309	1.406848	15.295838
H	6.640332	1.394175	16.471426
O	1.194664	3.887560	20.733143
H	1.312402	4.823281	20.513594
H	0.691413	3.979509	21.551071
O	6.839004	5.239521	15.772875
H	7.141595	4.569201	15.058685
H	7.634784	5.675735	16.179672
O	5.200976	3.158458	19.399534
H	4.623039	3.255669	20.254221
H	4.858409	3.867387	18.726091
O	5.147824	14.692102	17.201855
H	5.733420	15.159380	16.515003
H	5.705815	13.893982	17.548660
O	6.026227	13.265131	20.767191
H	6.722242	11.710983	21.678209
H	6.802021	13.927318	21.089380
O	11.059956	0.818870	19.717751
H	10.106525	0.859543	19.473442
H	11.070133	1.067029	20.760258
O	10.860591	13.760074	17.923578
H	11.094648	14.186352	17.062033
H	10.156027	14.397190	18.162628
O	7.644867	1.886797	17.999840
H	6.859788	2.283048	18.476135
H	7.752526	1.002320	18.441776
O	1.914262	0.169854	14.286749
H	2.146749	-0.655283	14.745108
H	2.567776	3.454979	15.509604
H	1.054301	0.451308	14.617699
O	4.608585	5.137526	17.438776
H	5.421509	5.176148	16.788103
H	3.799503	4.967667	16.934372
O	2.493850	4.422955	15.782234
H	1.637428	4.529450	16.202806
O	8.763365	9.827454	16.857552
H	9.349161	10.302641	17.492531
H	9.292946	9.314971	16.183641
O	2.373095	13.790547	16.566448
H	3.307202	14.000729	16.764685
H	2.182184	12.892702	16.948866
O	0.290480	-0.354070	17.376665
H	1.120788	-0.830075	17.438440
H	0.310113	0.187730	18.238935
O	8.124362	3.135253	14.422694
H	7.533510	2.486244	14.903645

H	8.939800	3.106731	15.026705
O	6.518270	12.730528	18.232038
H	7.163039	12.093402	17.935949
H	6.419658	12.889705	19.208982
O	1.435227	11.273289	17.880579
H	1.068619	11.776404	18.647509
H	2.323329	10.993020	18.176420
O	8.016544	3.353786	21.115591
H	7.623888	4.235301	22.866304
H	7.385256	3.300406	20.399971
H	4.401767	7.406367	19.627180
O	3.688080	1.836191	15.595949
H	3.665133	1.636459	16.585421
H	2.932058	1.237704	15.273623
O	1.579569	1.122737	20.469582
H	2.299451	1.228408	19.802723
H	1.260852	2.020487	20.630930
O	3.785146	10.929398	25.454315
H	3.335509	11.812650	25.594810
H	4.512307	11.384425	24.899729
O	5.330341	-0.607537	24.261644
H	4.832571	-0.168367	23.449842
H	5.790709	0.125798	24.688164
O	9.736671	3.127511	16.538338
H	9.032615	2.692078	17.036459
H	9.599736	4.185116	16.719772
O	10.054199	6.600153	19.659880
H	11.035387	3.904085	19.744606
H	9.341371	7.098083	20.174881
O	5.168938	8.098540	26.065407
H	5.246966	7.652803	25.207253
H	4.691051	8.934587	25.883341
O	10.476370	11.077797	18.363997
H	10.362264	12.051022	18.195623
H	9.176088	12.462900	24.283951
O	8.488023	14.963271	18.851601
H	8.471293	14.806167	19.915489
H	7.974342	14.209939	18.463776
O	11.649389	11.109966	21.177780
H	11.387547	10.601578	20.365662
H	12.135829	11.889712	20.669052
O	10.372892	4.264258	20.397112
H	8.967330	3.781806	20.752237
O	10.912302	1.730261	22.235708
H	11.842047	1.375389	22.928198
H	10.967712	2.771288	22.270060
O	12.832984	12.840932	19.607166
H	13.149729	13.413139	20.305908
H	12.293966	13.463581	19.043268
O	5.559299	7.166875	21.268391
H	6.496832	7.029378	21.204578
H	5.506778	7.948246	21.936338
H	5.182339	13.485250	21.193647

O	7.287052	10.189145	25.316198
H	8.077139	9.730392	24.996117
H	6.614256	9.471636	25.596235
O	7.151549	10.798442	21.705057
H	11.343210	10.822351	17.978199
H	7.011986	10.444650	20.746305
O	1.660136	6.648833	23.464687
H	6.804102	8.156698	18.642603
H	1.524405	6.817829	22.497429
O	8.254804	14.501227	21.423262
H	8.281557	15.509306	21.981838
H	1.504670	7.460800	23.938410
O	13.648086	4.508832	17.506279
H	13.069132	3.713438	17.837732
H	14.200040	4.755276	18.293087
O	11.905290	6.843675	16.937532
H	12.149687	7.693681	17.354134
H	12.536134	6.123362	17.267611
O	10.730952	-0.579880	15.383066
H	10.016006	0.005414	15.117678
H	11.539462	0.030388	15.278294
O	12.500064	9.454279	17.816719
H	12.604434	8.959114	18.665789
H	13.450409	9.631564	17.499121
O	1.647465	6.532073	20.479067
H	0.778195	6.970154	20.291842
H	2.211186	6.970947	19.750483
O	5.312244	9.040960	23.025562
H	5.994518	9.784161	22.904362
H	4.438950	9.374997	22.685259
O	12.569198	7.593858	19.861032
H	12.398868	8.115029	20.691181
H	11.691530	7.136806	19.654699
O	13.799397	9.391455	22.041306
H	13.072083	10.064788	21.756203
H	14.621746	9.657455	21.493561
O	1.428764	2.095853	24.032324
H	2.227264	1.957522	23.476494
H	1.346990	3.092453	24.056862
O	8.428978	1.111435	22.715355
H	9.417179	1.089767	23.010223
H	8.428053	1.675967	21.987770
O	6.858850	1.554168	24.974119
H	7.139131	1.353416	24.047373
H	6.700113	2.198100	25.546288
O	3.818689	7.267437	18.819481
H	4.653167	10.041098	18.752354
H	4.182298	6.376593	18.305067
O	9.099722	12.826133	25.183737
H	8.769915	12.095624	25.777939
H	13.881540	16.306129	23.828897
O	14.164541	14.385325	21.511042
H	14.387250	15.116858	20.912045
H	13.456079	14.897881	22.188452
O	1.282287	9.309207	24.691000
H	2.107953	9.730258	24.947798
H	0.998479	9.636074	23.792265
O	3.135148	13.052506	21.820677
H	2.420637	13.660984	22.123909
H	3.696958	12.887486	22.673685
O	9.586464	12.214849	22.251053
H	10.351799	11.845026	21.672455
H	8.830186	11.569127	22.058960
O	13.595933	4.457529	23.315926
H	13.815005	5.339623	23.729572
H	12.574731	4.399646	23.396749
O	2.780288	10.127250	21.298693
H	3.138018	10.266708	20.387991
H	2.629921	11.063047	21.595728
O	6.684335	9.096511	18.714260
H	10.123602	5.673604	20.139416
H	7.337455	9.369405	18.004044
O	13.008307	15.922171	23.649132
H	8.864175	13.732250	21.772202
H	12.713807	15.180331	24.184589
O	3.665503	10.014597	18.896793
H	3.531128	9.013004	18.672207
O	3.099476	13.900579	25.050508
H	2.600203	14.734967	24.909075
H	4.059594	14.151087	24.759926
O	12.220418	13.740050	24.934692
H	11.653295	13.386112	24.358200
H	12.113975	13.144942	25.693075
O	4.193841	5.704019	23.206472
H	3.361912	6.211652	23.352703
H	4.860977	6.245754	22.746681
O	3.683439	3.411664	21.635950
H	2.737406	3.626413	21.254805
H	3.910393	4.216909	22.170448
O	12.931617	1.157081	15.420757
H	13.143930	0.516733	16.168209
H	12.769940	2.008782	15.938615
O	5.939925	12.287613	23.692629
H	5.844117	13.241559	23.939762
H	6.529016	12.031675	24.467354
O	3.851250	0.870401	18.748693
H	4.472800	0.147384	18.394573
H	4.482035	1.598886	19.066261
O	10.922446	4.298077	22.791985
H	10.805129	4.283024	21.566355
H	10.580091	5.126370	23.092678
O	5.354292	4.188339	25.581024
H	5.414744	5.058944	26.024044
H	6.191862	4.225560	25.009468
O	9.361609	5.794937	17.066647
H	10.353367	6.250021	17.081842

H	9.326258	5.722082	18.099049

G2 (with water slab)

I	10.390437	8.948436	23.555252
Cl	8.899803	6.981750	23.581207
O	7.854981	5.018574	23.461359
H	8.644655	4.790241	24.260262
O	11.701509	3.008296	18.583467
H	11.711988	2.045736	18.957750
H	10.917460	2.917981	17.907215
O	3.652500	1.137302	22.749924
H	4.036469	1.955755	22.296761
H	3.064481	0.784734	21.981026
O	6.194283	0.989407	15.462164
H	5.406448	1.599992	15.329527
H	6.682460	1.304688	16.269951
O	1.273536	3.613248	20.948008
H	1.335346	4.563047	20.616247
H	0.744221	3.794049	21.743696
O	6.967520	5.433324	15.718292
H	7.040624	4.570059	15.309893
H	7.840127	5.696946	16.161535
O	5.616743	3.395825	19.226416
H	5.011172	3.434592	19.976004
H	5.332915	4.110772	18.616144
O	5.064121	14.652613	17.139900
H	5.566150	15.169923	16.440800
H	5.690448	13.984594	17.462164
O	5.882288	13.158124	20.711369
H	6.500306	11.555293	21.728016
H	6.636330	13.712903	21.063707
O	10.990092	0.890339	19.932507
H	10.188100	0.457118	19.550545
H	10.806572	1.062601	20.884312
O	10.830024	13.633759	17.822891
H	10.940445	14.042233	16.923016
H	10.022110	14.083875	18.169468
O	7.298085	1.750516	17.830660
H	6.607802	2.165597	18.379690
H	7.481835	0.895928	18.380962
O	1.806888	0.066461	14.459404
H	1.883101	-0.654962	15.106145
H	2.648571	3.808729	15.529480
H	0.983099	0.494116	14.683825
O	4.785109	5.257185	17.356007
H	5.490427	5.353095	16.614956
H	3.812504	5.064730	16.966862
O	2.455106	4.705225	15.982738
H	1.541255	4.621587	16.434210
O	8.477616	9.568106	17.350986
H	9.143291	10.276874	17.671278
H	8.429810	9.611861	16.372877
O	2.579262	13.703060	16.611984
H	3.548027	13.963496	16.664528
H	2.431645	12.861376	17.156433
O	0.644637	15.381435	17.481812
H	1.389601	14.715948	17.225904
H	0.738049	15.521010	18.488228
O	8.115818	2.885604	14.278859
H	7.574092	2.062974	14.174157
H	8.822311	2.752652	14.965294
O	6.581019	12.684865	18.210608
H	6.824184	11.779435	18.067436
H	6.408864	12.886500	19.166496
O	1.414610	11.512005	18.025095
H	0.990813	12.086153	18.676516
H	2.022469	10.974673	18.538162
O	8.075768	3.317194	21.409843
H	7.891541	4.236591	22.636507
H	7.270356	3.477424	20.908621
H	4.634850	7.208954	19.932810
O	3.695487	2.072199	15.452266
H	3.456257	1.828287	16.382254
H	3.031103	1.535014	14.941417
O	1.545425	1.058071	20.276115
H	2.356388	0.987441	19.697538
H	1.329458	2.041148	20.471893
O	3.876520	10.760917	24.967216
H	3.508025	11.672727	25.154829
H	4.706062	11.087171	24.448870
O	5.452455	-0.378262	24.375074
H	5.092673	0.006393	23.535669
H	5.921285	0.417857	24.735184
O	9.665093	3.024783	16.881012
H	8.868255	2.679164	17.354134
H	9.609912	4.012793	16.912714
O	10.045894	6.654094	19.788460
H	10.654199	3.698043	19.923510
H	9.385169	6.858056	20.505438
O	5.362242	8.073673	25.843599
H	5.336271	7.884416	24.900902
H	4.542508	8.572517	26.046455
O	10.488354	11.106810	18.374023
H	10.718344	12.031417	18.033327
H	9.131825	12.867355	23.994894
O	8.393718	14.818609	18.722216
H	8.321376	14.814804	19.802145
H	8.023206	13.961687	18.285404
O	11.601951	10.791660	20.934561
H	10.987159	10.653761	20.180035
H	12.134714	11.487170	20.520784
O	10.109537	4.072224	20.588606
H	9.116449	3.745706	20.821842
O	10.912076	1.625823	22.443508
H	11.843700	0.807885	22.814411

H	11.173352	2.418970	22.844257	O	3.847168	7.175971	19.290344
O	13.035204	12.818519	19.679096	H	4.807875	10.278194	18.727400
H	13.208226	13.410670	20.475054	H	4.061638	6.564448	18.573586
H	12.438773	13.360447	19.122665	O	9.267025	12.995468	24.959366
O	5.634488	7.182962	21.006477	H	8.689237	12.327052	25.404434
H	6.553072	6.922300	21.016405	H	13.716029	16.310444	23.619230
H	5.539243	7.724705	21.836693	O	0.799274	14.389856	21.466248
H	5.084098	13.524431	21.138889	H	0.949459	15.270373	20.991650
O	7.388146	10.107102	25.305197	H	0.207596	14.748039	22.131584
H	7.866597	9.493413	24.748039	O	1.191574	9.208233	24.551437
H	6.662132	9.547775	25.643661	H	1.963622	9.787212	24.454716
O	6.965439	10.739158	21.850334	H	0.599510	9.389593	23.776281
H	11.216997	10.481954	18.121008	O	3.237409	13.007790	21.758501
H	7.066863	10.365898	20.911963	H	2.509801	13.301363	21.230581
O	1.630171	6.924575	23.555792	H	2.949272	13.413901	22.651529
H	6.015610	8.723309	18.756693	O	9.199657	12.212847	22.460514
H	1.521158	7.262970	22.641674	H	10.084816	11.918907	22.125624
O	8.153227	14.578979	21.369314	H	8.551896	11.545400	22.104351
H	8.064077	15.501803	21.883217	O	13.601066	4.563135	22.870689
H	1.516293	7.810280	24.141514	H	13.899767	5.390884	23.224140
O	13.611224	4.523559	17.713818	H	12.566493	4.544125	23.071699
H	12.790585	3.851704	17.919144	O	3.051774	10.403145	21.493996
H	14.194263	4.316692	18.477610	H	3.517709	10.191936	20.615200
O	11.474201	7.039127	16.839130	H	3.212939	11.376523	21.558352
H	11.698713	8.004598	16.862381	O	6.831429	8.912738	19.165377
H	12.260863	6.519641	17.240974	H	10.282296	5.706374	20.051430
O	10.773232	-0.706871	15.331916	H	7.547863	8.895235	18.506319
H	9.896839	-0.303859	15.361976	O	12.786951	15.785979	23.298901
H	11.410623	0.011421	15.491872	H	8.703183	14.026657	21.932432
O	12.671792	9.641435	17.860579	H	12.633386	15.187081	24.043169
H	12.981200	8.877551	18.398849	O	3.937582	9.794520	18.649460
H	13.347938	10.390680	17.874725	H	3.914395	8.800946	18.884037
O	1.523353	6.231002	20.633015	O	3.058889	13.769706	24.765604
H	0.761768	6.584849	20.120886	H	2.236550	14.302789	24.850868
H	2.325353	6.523258	20.052830	H	3.789682	14.348869	24.618580
O	5.111012	9.078038	22.978466	O	12.227399	13.495197	24.788094
H	5.759690	9.739690	22.724884	H	11.351363	13.294385	24.475229
H	4.272368	9.585544	22.751232	H	12.062772	13.831583	25.707317
O	12.816464	7.774223	19.761946	O	3.937597	5.432330	23.094780
H	13.010464	8.378830	20.440359	H	3.104167	5.891085	23.401043
H	11.824965	7.580450	20.056532	H	4.233551	6.047560	22.384584
O	13.900562	9.289630	21.736229	O	4.032744	3.381665	21.551910
H	13.183634	9.855799	21.345968	H	3.117666	3.364822	21.256866
H	14.703734	9.756491	21.469343	H	3.960265	4.160094	22.257193
O	1.321340	1.922330	24.065750	O	12.691803	1.179893	15.641364
H	2.189308	1.583111	23.674814	H	13.060848	0.751149	16.464149
H	1.187351	2.722163	23.486023	H	12.585096	2.160971	15.846056
O	8.322064	1.257623	22.963741	O	6.083215	12.370018	23.772869
H	9.385558	1.261233	22.880068	H	6.028890	13.266353	24.168072
H	7.985207	1.952593	22.318504	H	6.684655	11.831477	24.366434
O	6.674813	1.874340	25.042351	O	3.731135	1.001532	18.576338
H	7.505941	1.918971	24.573088	H	4.348989	0.302726	18.240185
H	6.035752	2.577567	24.471853	H	4.225173	1.816675	18.611242

O	11.049229	4.241151	22.906181
H	10.712477	4.294284	21.952372
H	10.452749	4.667604	23.511295
O	5.729603	4.441187	25.450356
H	5.571652	4.833646	26.338562
H	5.106875	4.853724	24.779381
O	9.332500	5.651130	16.963438
H	10.163580	6.155989	16.577543
H	9.483844	6.006525	17.896156

G3 (with water slab)

I	9.566000	7.520000	23.638000
Cl	7.130001	6.540000	22.777002
O	5.732000	5.528000	22.320999
H	5.282000	5.372001	23.209999
O	12.004000	4.419000	15.595001
H	11.716001	4.637000	16.525000
H	11.112001	4.615000	15.152000
O	1.508000	0.464000	21.456001
H	1.528000	1.370000	21.075001
H	1.906000	-0.092000	20.756001
O	4.337000	0.611000	14.975000
H	3.745000	0.873000	14.272001
H	4.713000	1.438000	15.396000
O	12.345001	2.587000	21.275999
H	11.546000	2.685000	20.728001
H	12.101000	2.993000	22.180002
O	5.498000	2.722000	16.409000
H	5.532000	2.542000	17.386999
H	6.446000	2.572000	16.124001
O	2.679000	2.291000	18.627001
H	2.180000	2.648000	19.408001
H	2.714000	2.942000	17.871000
O	3.309000	14.356000	16.608999
H	3.761000	14.911000	15.947000
H	3.950000	13.759001	17.056000
O	3.743000	12.456000	20.613001
H	4.411000	11.014000	20.723000
H	4.029000	13.091000	21.365002
O	10.149000	0.079000	19.212000
H	11.137000	-0.143000	19.211000
H	9.879001	-0.490000	19.983000
O	8.424000	0.064000	17.188000
H	9.083000	0.137000	17.969000
H	7.602001	-0.232000	17.684999
O	5.157000	2.087000	18.969000
H	4.125000	2.094000	18.899000
H	5.410000	1.119000	18.948000
O	11.927000	0.203000	13.687000
H	11.832000	-0.659000	14.097000
H	1.094000	3.305000	14.530000
H	11.752001	0.895000	14.387001
O	2.887000	4.510000	16.761999
H	3.775000	4.223001	16.364000
H	2.208000	4.420000	15.992001
O	1.130000	4.298000	14.725000
H	0.211000	4.447000	15.075001
O	8.114000	11.410000	18.818001
H	8.106000	11.731000	19.739000
H	8.941000	11.693000	18.351999
O	0.777000	12.906000	15.825000
H	1.576000	13.495000	15.708000
H	1.121000	12.378000	16.677000
O	11.565000	13.975000	16.177000
H	12.559000	13.855000	16.143000
H	11.452001	14.825000	16.622999
O	8.899000	0.110000	14.247000
H	9.831000	-0.012000	13.995000
H	8.760000	-0.453000	15.018000
O	5.106000	12.852000	18.285000
H	5.780000	12.070001	18.313000
H	4.489000	12.779000	19.124001
O	1.464000	11.789000	18.162001
H	0.670000	11.832000	18.749001
H	1.852000	10.906000	18.391001
O	6.778000	3.715000	20.341999
H	6.487000	4.455000	20.892000
H	5.967000	3.292000	19.940001
H	2.751000	6.330000	19.434999
O	1.200000	1.073000	14.436001
H	1.296000	0.848000	15.371000
H	0.374000	0.687000	14.168000
O	12.859000	14.773000	19.686001
H	13.607000	14.991000	19.080000
H	12.933000	15.373000	20.438002
O	2.067000	9.531000	26.313000
H	2.658000	10.236000	25.985001
H	1.236000	9.831000	25.892000
O	2.986000	-0.064000	23.740999
H	2.482000	0.078000	22.889002
H	3.537000	0.787000	23.781002
O	9.393000	4.813000	15.015000
H	9.050000	4.005000	15.523000
H	8.982000	5.548000	15.553001
O	9.584000	5.314000	21.153000
H	9.727000	1.747000	19.356001
H	9.770000	6.099000	21.666000
O	4.127000	7.942000	25.290001
H	3.317000	8.330000	25.669001
H	4.045000	8.134000	24.304001
O	7.724000	11.963000	21.572001
H	6.917000	12.394000	21.837999
H	8.478000	9.392001	23.747999
O	6.347000	15.075001	18.924000
H	6.653000	15.119000	19.818001

H	5.885000	14.167000	18.791000
O	10.726000	11.191000	21.433001
H	10.393000	10.229000	21.368000
H	10.684001	11.317000	22.399002
O	9.856000	2.701000	19.549000
H	9.017000	2.891000	19.896999
O	9.653000	14.192001	21.403000
H	10.405000	14.156001	22.030001
H	9.287000	13.255001	21.405001
O	10.616000	11.816000	17.767000
H	11.210000	11.993000	18.513000
H	10.931000	12.455000	17.035000
O	3.452000	6.266000	20.950001
H	4.391000	6.080000	21.065002
H	3.424000	7.190000	21.336000
H	2.779000	12.231000	20.785999
O	6.137000	10.090001	25.497999
H	5.364000	10.558000	25.106001
H	5.755000	9.168000	25.539000
O	4.620000	10.028001	20.670002
H	7.829000	11.335000	22.361000
H	5.527000	9.771000	20.350000
O	13.307000	5.755000	22.553001
H	7.184000	8.348000	19.228001
H	13.032000	5.924000	21.594999
O	7.067000	15.263000	21.709999
H	7.153000	16.229000	21.916000
H	13.090000	6.533000	23.084000
O	11.306001	4.712000	18.214001
H	11.056000	3.868000	18.643999
H	11.985000	5.132000	18.785000
O	9.391000	6.728000	17.518000
H	10.063000	7.387000	17.900000
H	9.863000	5.890000	17.628000
O	8.307000	2.272000	15.787000
H	8.499001	1.558000	16.535999
H	8.401000	1.688000	14.977001
O	11.406000	8.524000	18.799999
H	10.773000	8.752000	19.568001
H	11.644000	9.380000	18.445999
O	13.071000	6.128000	19.683001
H	12.574000	6.915000	19.364000
H	13.958000	6.152000	19.202999
O	3.360000	8.798000	22.566002
H	3.919000	9.285000	21.860001
H	2.375000	8.944000	22.209000
O	9.655000	8.891000	21.047001
H	9.581000	8.275000	21.818001
H	8.713000	8.933000	20.653999
O	12.653001	8.585000	23.391001
H	11.694001	8.567000	23.181000
H	13.104000	8.675000	22.541000
O	-0.503000	1.114000	24.202999
H	0.204000	1.076000	23.509003
H	-0.720000	1.984000	24.510000
O	6.938001	2.233000	22.607000
H	7.651000	2.694000	23.090000
H	6.965001	2.710000	21.694000
O	4.412000	2.117000	23.754002
H	5.304000	2.167000	23.221001
H	4.517001	2.887000	24.326000
O	2.051000	6.383000	18.691000
H	3.195000	9.459000	19.657000
H	2.393000	5.825000	17.951000
O	8.090000	10.314000	23.773001
H	7.378000	10.129000	24.466999
H	11.819001	15.142000	23.900002
O	12.529001	12.175000	19.886999
H	12.560000	13.151000	19.884001
H	11.867000	11.816000	20.562000
O	13.167000	10.604000	25.069000
H	13.516001	11.274000	24.472000
H	12.950000	9.732000	24.491001
O	1.260000	11.909000	21.531000
H	0.470000	12.035001	20.875000
H	1.038000	12.392000	22.415001
O	4.717000	13.770000	22.595001
H	4.277000	14.432000	23.173000
H	5.527000	14.272000	22.287003
O	11.876000	3.817000	23.716000
H	12.397001	4.596000	23.351000
H	10.922001	3.966000	23.845001
O	0.995000	9.300000	21.594999
H	1.265000	9.155000	20.667999
H	1.013000	10.288000	21.646999
O	7.229001	9.164000	19.697001
H	8.882000	5.601000	20.496002
H	7.452000	9.950000	19.082001
O	11.659000	14.245001	23.627003
H	7.968000	14.882000	21.862000
H	11.047000	13.695000	24.201000
O	2.409000	9.209000	19.150000
H	2.571000	8.311000	18.875999
O	0.691000	13.264001	23.604000
H	-0.176000	13.809000	23.726002
H	1.355000	13.837000	24.003000
O	10.202000	11.904000	24.382000
H	9.389000	11.331000	24.351999
H	10.878001	11.295000	24.787003
O	2.334000	4.551000	22.681000
H	1.465000	5.016000	22.773001
H	2.787000	5.148000	22.041000
O	1.361000	2.967000	20.662001
H	0.324000	2.962000	20.770000
H	1.730000	3.485000	21.427000
O	11.333000	1.791000	15.903000

H	10.418000	1.980000	15.593000
H	11.807000	2.651000	15.683001
O	3.646000	11.251000	24.368000
H	3.886000	12.093000	23.938000
H	3.466000	10.579000	23.670002
O	1.588000	-0.249000	18.353001
H	2.131000	-0.739000	17.652000
H	1.793000	0.755000	18.280001
O	8.990000	3.851000	23.368000
H	9.260000	4.226000	22.451000
H	8.611000	4.650000	23.813000
O	4.187000	5.251000	24.576000
H	4.167000	6.193000	24.931000
H	3.315000	5.068000	24.091999
O	7.742000	6.148000	19.417999
H	8.298001	6.583001	18.709000
H	7.395000	5.347000	19.030001
